# Integration of Genetic and Process Engineering for Optimized Rhamnolipid Production Using *Pseudomonas putida*

**DOI:** 10.3389/fbioe.2020.00976

**Published:** 2020-08-20

**Authors:** Till Tiso, Nina Ihling, Sonja Kubicki, Andreas Biselli, Andreas Schonhoff, Isabel Bator, Stephan Thies, Tobias Karmainski, Sebastian Kruth, Anna-Lena Willenbrink, Anita Loeschcke, Petra Zapp, Andreas Jupke, Karl-Erich Jaeger, Jochen Büchs, Lars M. Blank

**Affiliations:** ^1^iAMB – Institute of Applied Microbiology, ABBt – Aachen Biology and Biotechnology, RWTH Aachen University, Aachen, Germany; ^2^Bioeconomy Science Center (BioSC), Forschungszentrum Jülich GmbH, Jülich, Germany; ^3^Chair of Biochemical Engineering (AVT.BioVT), RWTH Aachen University, Aachen, Germany; ^4^Institute of Molecular Enzyme Technology, Heinrich-Heine-Universität Düsseldorf, Düsseldorf, Germany; ^5^Fluid Process Engineering (AVT.FVT), RWTH Aachen University, Aachen, Germany; ^6^Institute of Energy and Climate Research – Systems Analysis and Technology Evaluation (IEK-STE), Forschungszentrum Jülich GmbH, Jülich, Germany; ^7^Institute of Bio- and Geosciences IBG 1: Biotechnology, Forschungszentrum Jülich GmbH, Jülich, Germany

**Keywords:** rhamnolipids, *Pseudomonas putida* KT2440, synthetic biology, metabolic engineering, oxygen transfer rate, liquid–liquid extraction, life cycle assessment, environmental impact

## Abstract

Rhamnolipids are biosurfactants produced by microorganisms with the potential to replace synthetic compounds with petrochemical origin. To promote industrial use of rhamnolipids, recombinant rhamnolipid production from sugars needs to be intensified. Since this remains challenging, the aim of the presented research is to utilize a multidisciplinary approach to take a step toward developing a sustainable rhamnolipid production process. Here, we developed expression cassettes for stable integration of the rhamnolipid biosynthesis genes into the genome outperformed plasmid-based expression systems. Furthermore, the genetic stability of the production strain was improved by using an inducible promoter. To enhance rhamnolipid synthesis, energy- and/or carbon-consuming traits were removed: mutants negative for the synthesis of the flagellar machinery or the storage polymer PHA showed increased production by 50%. Variation of time of induction resulted in an 18% increase in titers. A scale-up from shake flasks was carried out using a 1-L bioreactor. By recycling of the foam, biomass loss could be minimized and a rhamnolipid titer of up to 1.5 g/L was achieved without using mechanical foam destroyers or antifoaming agents. Subsequent liquid–liquid extraction was optimized by using a suitable minimal medium during fermentation to reduce undesired interphase formation. A technical-scale production process was designed and evaluated by a life-cycle assessment (LCA). Different process chains and their specific environmental impact were examined. It was found that next to biomass supply, the fermentation had the biggest environmental impact. The present work underlines the need for multidisciplinary approaches to address the challenges associated with achieving sustainable production of microbial secondary metabolites. The results are discussed in the context of the challenges of microbial biosurfactant production using hydrophilic substrates on an industrial scale.

## Introduction

Rhamnolipids are versatile anionic glycolipid biosurfactants produced by different bacteria (Germer et al., 2020). Discussed applications range from fine chemicals for pharmaceuticals and cosmetics to bulk chemicals like detergents, bioremediation agents, and enhanced oil recovery agents. Diverse patents have been filed for rhamnolipid production and their application (Tiso et al., 2017a), and recently large-scale industrial production was announced.

Rhamnolipids consist of 3-(3-hydroxyalkanoyloxy)alkanoate (HAA), with hydrophobic acyl chains linked to a hydrophilic moiety formed by one or two molecules of rhamnose. The three surface active compounds HAAs and mono- and di-rhamnolipids show differences in physiochemical parameters like the critical micelle concentration (Tiso et al., 2017b). The biosynthesis of rhamnolipids starts with the formation of HAA from two β-hydroxy fatty acids by the enzyme RhlA (Déziel et al., 2003). The substrate spectra of different RhlA enzymes are responsible for the chain-length spectrum of the final biosurfactant (Dulcey et al., 2019; Germer et al., 2020). Subsequently, rhamnose units are attached to the HAA by activity of RhlB and RhlC, respectively, to form mono- and di-rhamnolipids (Ochsner et al., 1994; Rahim et al., 2001). All three enzymes are able to act independently (Wittgens et al., 2017). The mono-rhamnolipid biosynthesis encoding genes *rhlA* and *rhlB* are joint to one transcriptional unit in all producer strains known so far (Tiso et al., 2017a). The precursors (hydroxy fatty acids and rhamnose as activated conjugates) are provided by the cell’s central metabolism (Tiso et al., 2016).

To avoid issues connected to the general pathogenicity of *Pseudomonas aeruginosa*, which is the best-characterized rhamnolipid-producing species, and circumvent complex natural regulation mechanisms for the production of these secondary metabolites, *Pseudomonas putida* has very successfully been developed during the last decade to a recombinant rhamnolipid production platform applying the toolbox of synthetic biology. It allows for high-level production (Beuker et al., 2016a) and tailoring of the product spectrum (Tiso et al., 2017b). In this context, the development of plasmids conferring a strong expression of the biosynthetic operons in the host organism (Beuker et al., 2016a; Wigneswaran et al., 2016) has been combined with detailed characterization of the cell factory (Tiso et al., 2016), elimination of concurring metabolic pathways (Wittgens et al., 2011), and expansion of the spectrum of feedable (Arnold et al., 2019; Wang et al., 2019; Bator et al., 2020b) renewable resource.

The use of inducible (genome-integrated or plasmid-based) promoter systems enables an easily applicable option for process control. In addition, the use of an inducible system offers the possibility of controlling production by, for example, decoupling it from growth. By variation of inducer concentration and time of induction, recombinant protein expression can be tuned and optimized (Wandrey et al., 2016; Mühlmann et al., 2017). For *Escherichia coli*, monitoring of the oxygen transfer rate (OTR) or the scattered light signal was demonstrated to be a useful tool for optimization of recombinant protein production in small scale (Ladner et al., 2017; Ihling et al., 2020). In addition, non-invasive online monitoring of the OTR in shake flasks combined with analysis of offline-determined culture parameters can be used for a detailed assessment of cultivation conditions and whole-cell performance. As process development moves forward, scale-up from shake flask to stirred tank usually needs to be tackled.

For separation and purification of rhamnolipids from fermentation supernatants, various approaches are reported in literature. These include liquid–liquid extraction (Syldatk et al., 1985; Schilling et al., 2016), pH-shift precipitation with subsequent solid–liquid extraction (Invally et al., 2019), membrane filtration (Witek-Krowiak et al., 2011), chromatographic separation (Reiling et al., 1986), and foam fractionation, optionally extended by adsorption columns (Siemann-Herzberg and Wagner, 1993; Anic et al., 2018). To design reliable and efficient downstream processes, in-depth knowledge with regard to the influence of process parameters like detrimental interphase formation on the process performance is mandatory.

Production and application of biosurfactants is claimed to be more sustainable compared to conventional fossil-based surfactants. Sustainability-related discussions and developments, which find expression in political statements of intent (BMU, 2019), the “green deal” of the EU, or the most recently strongly grown environmental movement (Brändlin, 2019), underline the importance of a thoughtful and analyzed implementation of new products and processes. Beyond that, the definition of nationally and globally defined criteria for sustainability (United Nations [UN], 2015; Die Bundesregierung, 2018) clarifies the significance of its evaluation. As one of three dimensions, the environmental sustainability forms the most obvious one and is therefore considered below (Finkbeiner et al., 2010). Looking at the brief history of LCA for (bio-)surfactants, it is remarkable that there is only a limited number of such studies. Examples can be seen in Adlercreutz et al. (2010) where alkanolamides (bio-based surfactants) were evaluated. Sophorolipid and rhamnolipid production in a gate-to-gate system was subject to investigations in Kopsahelis et al. (2018), while Baccile et al. (2017) evaluated the production of sophorolipids in a cradle-to-gate and cradle-to-grave system. Further studies can be found with regard to conventional surfactants (Guilbot et al., 2013; CESIO/ERASM, 2017) or detergents (Zah and Hischier, 2007).

**FIGURE 1:**
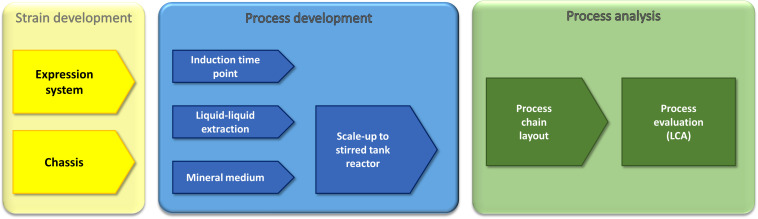
Multidisciplinary approach for the optimization and evaluation of a recombinant rhamnolipid production strategy. Our approach comprises molecular biology, metabolic engineering, bioprocess engineering, fluid process engineering, and system analysis and technology evaluation (LCA). The parameters that were used by the respective approaches as criteria to evaluate the success are written in the sketched funnels.

In this work, we use a holistic approach based on strain development and process optimization to enhance recombinant rhamnolipid production (Figure 1). The strain development includes the engineering of an efficient expression cassette featuring high genetic stability, which is important for industrial processes as well as chassis optimization. The process optimization efforts are the identification of a suitable minimal medium, detailed investigation of the optimal induction time point, and, based on previous work, evaluation of the impact of an optimized fermentation medium on the performance of a liquid–liquid extraction. The gained insights allowed the development of a process chain, which was finally evaluated *via* LCA. As the final process would be run in a stirred tank reactor, the LCA was performed in small pilot scale (150-L fermentation volume). Integrating LCA while the process is still in an early stage of development favors the identification of process options that can directly be integrated in subsequent developments.

## Materials and Methods

### Bacterial Strains and Plasmids

Bacterial strains (Table 1) and plasmids (Table 2) used in this study are shown here. The used oligonucleotides are shown in Supplementary Table 1.

**TABLE 1:** Bacterial strains used in this work.

**Strain**	**Features**	**References**
**Strains used for cloning, amplification, and transfer of plasmids**		
*E. coli* DH5α	F^–^ Φ80*lacZ*ΔM15 Δ(*lacZYA*-*argF*) U169 *deoR*, *recA1*, *endA1*, *hsdR17* (rK−, mK_+_) *phoA supE44*λ*_max_*^–^ thi^–^1, *gyrA96*, *relA1*,	Hanahan, 1983
*E. coli* S17-1	*recA*, *thi*, pro, *hsdR*^–^*M*^+^ [RP4 2-Tc^*R*^:Mu:Km^*R*^Tn7] Tp^*R*^ Sm^*R*^	Simon et al., 1983
*S. cerevisiae* VL6-48	MATa, his3-Δ200, trp1-Δ1, ura3–52, lys2, ade2–101, met14	Kouprina et al., 1998
*E. coli* DH5αλpir	λpir lysogen of DH5α; host for *oriV*(R6K) vectors	de Lorenzo and Timmis, 1994
*E. coli* PIR2	F^–^, Δ*lac169*, *rpoS*(Am), *robA1*, *creC510*, *hsdR514*, *endA*, *recA1*, *uidA*(Δ*Mlu*I):pir; host for oriV(R6K) vectors	Thermo Fisher Scientific, Waltham, MA, United States
*E. coli* HB101 pRK2013	Sm^*R*^, *hsdR*-*M*^+^, *proA2*, *leuB6*, *thi-1*, *recA*; harboring plasmid pRK2013	Ditta et al., 1980
*E. coli* DH5α pYTSK01K_0G7	DH5α harboring plasmid pYTSK01K_0G7	This study
*E. coli* DH5α pVLT33-PA_*rhlABC*	DH5α harboring plasmid pVLT33-PA_*rhlABC*	Wittgens et al., 2017
*E. coli* DH5α pBNT Km	DH5α harboring plasmid pBNTmcs(t)Km	This study
*S. cerevisiae* VL6-48 pYTSK10K_0G7_*rhlAB*	VL6-48 harboring plasmid pYTSK10K_0G7_*rhlAB*	This study
*E. coli* DH5α pYTSK10K_1G7_*rhlAB*	DH5α harboring plasmid pYTSK10K_1G7_*rhlAB*	This study
*E. coli* DH5α pRhon5Hi-2-*eyfp*	DH5α harboring plasmid pRhon5Hi-2-*eyfp*	Troost et al., 2019
*S. cerevisiae* VL6-48 pYTSK40K_1G7_*rhlAB*	VL6-48 harboring plasmid pYTSK40K_1G7_*rhlAB*	This study
*E. coli* DH5α pYTSK40K_0G7_*rhlAB*	DH5α harboring plasmid pYTSK40K_0G7_*rhlAB*	This study
*E. coli* S17-1 pYTSK40K_1G7_*rhlAB*	S17-1 harboring plasmid pYTSK40K_1G7_*rhlAB*	This study
*E. coli* DH5αλpir pSK02	DH5αλpir harboring Tn7 delivery vector pSK02 for chromosomal integration	Bator et al., 2020b
*E. coli* DH5α pSW-2	DH5α harboring plasmid pSW-2 encoding I-*Sce*I nuclease	Martinez-Garcia and de Lorenzo, 2011
*E. coli* DH5αλpir pTNS-1	DH5αλpir harboring plasmid pTNS-1	Choi et al., 2005
*E. coli* DH5αλpir pΔpha	DH5αλpir harboring plasmid pΔpha	Mato Aguirre, 2019
*E. coli* PIR2 pEMG-flag1	PIR2 harboring plasmid pEMG-flag1	Blesken et al., 2020
*E. coli* PIR2 pEMG-flag2	PIR2 harboring plasmid pEMG-flag2	Blesken et al., 2020
*E. coli* DH5αλpir pMaW03	DH5αλpir harboring plasmid pMaW03	This study
***P. putida* chassis strains**		
*P. putida* KT2440	Wild type	Bagdasarian et al., 1981
*P. putida* KT2440 Δflag	ΔPP_4328-PP_4344 and ΔPP_4351-PP_4397	Blesken et al., 2020
*P. putida* KT2440 ΔphaG	ΔPP_1408	This study
*P. putida* KT2440 Δpha	ΔPP_5003-PP_5008	Blesken et al., 2020
*P. putida* KT2440 Δ*phaG* Δpha	ΔPP_1408 and ΔPP_5003-PP_5008 (*phaC1ZC2DFI*)	This study
*P. putida* KT2440 Δflag Δpha	ΔPP_4328-PP_4344, ΔPP_4351-PP_4397 and ΔPP_5003-PP_5008	This study
***P. putida* strains used for biosurfactant production**		
*P. putida* KT2440 pPS05	KT2440 harboring plasmid pPS05	Tiso et al., 2016
*P. putida* KT2440 SK4	*rhlAB*, attTn7 integrated, P_14ffg_	This study
*P. putida* KT2440 *_*sal*_*mRL*^E^* SK40	*rhlAB*, *eYFP*, attTn7 integrated, P_*nagA*__a_/*nagR*, Gm^*R*^, *tnsABCD*	This study
*P. putida* KT2440 Δflag SK4	ΔPP_4328-PP_4344 and ΔPP_4351-PP_4397, *rhlAB*, attTn7 integrated, P_14ffg_	Blesken et al., 2020
*P. putida* KT2440 ΔphaG SK4	ΔPP_1408, *rhlAB*, attTn7 integrated, P_14ffg_	This study
*P. putida* KT2440 Δpha SK4	ΔPP_5003-PP_5008, *rhlAB*, attTn7 integrated, P_14ffg_	Blesken et al., 2020
*P. putida* KT2440 Δ*phaG* Δpha SK4	ΔPP_1408 and ΔPP_5003-PP_5008, *rhlAB*, attTn7 integrated, P_14ffg_	This study
*P. putida* KT2440 Δflag Δpha SK4	ΔPP_4328-PP_4344 and ΔPP_4351-PP_4397 and ΔPP_5003-PP_5008, *rhlAB*, attTn7 integrated, P_14ffg_	This study

**TABLE 2:** Plasmids used within this work.

**Name**	**Features**	**References**
pEMG-flag1	pEMG derivative for KO of ΔPP_4328-PP_4344	Blesken et al., 2020
pEMG-flag2	pEMG derivative for KO of ΔPP_4351-PP_4397	Blesken et al., 2020
pMaW03	pEMG derivative for KO of ΔPP_1408	This study
pΔpha	pEMG derivative for KO of ΔPP_5003-PP_5008	Mato Aguirre, 2019
pSK02	pBG derivative, Km^*R*^, Gm^*R*^, *oriV*(R6K), Tn7-L and Tn7-R, *rhlAB*, P_14ffg_	Bator et al., 2020b
pPS05	pBBR1 derivative, *rhlAB*, P_syn16_	Tiso et al., 2016
pRK2013	Km^*R*^, *oriV*(RK2/ColE1), mob^+^ tra^+^, helper plasmid for conjugation	Figurski and Helinski, 1979
pSW-2	Gm^*R*^, *oriV*(RK2), mob^+^, *xylS*, *Pm* → I-*Sce*I, plasmid for KO	Martinez-Garcia and de Lorenzo, 2011
pTNS-1	Ap^*R*^, *oriV*(R6K), mob^+^, *tns7A-D* for specific transposition, helper plasmid for Tn7 integration	Choi et al., 2005
pYTSK01K_0G7	YCp50-poly-Km^*R*^, Tn7-L, Gm^*R*^, *tns7A-D*, Tn7-R	GenBank Accession No. MT522186
pBNTmcs(t)Km	pBBR1 ori, Km^*R*^, P_*nagAa*,_ *nagR*	Verhoef et al., 2010
pVLT33-PA_*rhlABC*	pRSF1010 ori, oriT, Km*^*R*^*, EC*_lacI*, *P_*tac*__*PA*_rhlABC*	Wittgens et al., 2017
pRhon5Hi-2-*eyfp*	pBBR1 ori, oriT, Cm^*R*^, Km^*R*^, pelB, P*_*nifH5*_*_*eYFP*	Troost et al., 2019
pYTSK10K_0G7_*rhlAB*	YCp50-poly-Km*^*R*^*, Tn7-L, P*_*nagAa*_/nagR* + BCD2 + PA-*rhlAB*, Gm^*R*^, *tns7A-D*, Tn7-R	This study
pYTSK40K_1G7_*rhlAB*	YCp50-poly-Km*^*R*^*, Tn7-L, P*_*nagAa*_/nagR* + BCD2 + PA-*rhlAB*, *eYFP*, Gm^*R*^, *tns7A-D*, Tn7-R	This study

*KO, knockout.*

#### Construction of Deletion Mutants

Deletion mutants were obtained using the I-SceI-based system (Martinez-Garcia and de Lorenzo, 2011). Briefly, 500–700-bp upstream- and downstream-flanking regions of the target sites were amplified from the genomic DNA of *P. putida* KT2440 and cloned into the non-replicative pEMG vector. The resulting plasmids were transferred into *E. coli* DH5αλpir or PIR2 and conjugated into *Pseudomonas* strains via triparental mating using a streamlined method as outlined by Wynands et al. (2018). The pSW-2 plasmid, carrying the I-SceI restriction enzyme, was transformed to allow for the deletion of the gene locus of interest. Positive colonies sensitive for kanamycin were verified for targeted deletion by colony PCR. To obtain marker-free clones, the recombinant strains were cured of the pSW-2 plasmid by reinoculation in the LB medium without gentamicin and verified again by colony PCR. In this study, knockout mutants of the genes coding for the flagellar machinery [PP_4328–PP_4344 and PP_4351–PP_4397; a detailed overview about the components encoded by the target genes has been given by Martinez-Garcia et al. (2014)], the 3-hydroxyacyl-ACP:3-hydroxyacyl-CoA transacylase (*phaG*, PP_1408), and the complete operon for PHA synthesis (PP_5003–PP_5008) were created.

#### Construction of Rhamnolipid Producing Strains

Construction of *P. putida* KT2440 *_*sal*_*mRL^E^ SK40 was performed as follows: DNA fragments with homology arms of ca. 30 bp as building blocks for the subsequent assembly cloning were obtained by PCR as described before (Weihmann et al., 2020). Linearized plasmid backbones obtained by restriction endonuclease digestion with I-SceI or MauBI were dephosphorylated with FastAP (NEB, Frankfurt a. M, Germany, and Thermo Fisher Scientific GmbH, Waltham, MA, United States, respectively) according to the manufacturer’s instructions.

Vector assembly by recombinational cloning in uracil deficient yeast cells was performed as described before (Domröse et al., 2017; Weihmann et al., 2020). Competent cells of *Saccharomyces cerevisiae* VL6-48 were prepared according to Gietz and Schiestl (2007). Fragments encoding the NagR/P_nagAa_ expression system and proteins for mono-rhamnolipid biosynthesis (including a BCD2 translational coupler for *rhlA*) were amplified from pBNTmcs(t)Km and pVLT33_rhlABC using the oligonucleotides fw_CISL_NagR, rv_PnagAa_BCD2, fw_BCD2_PA-rhlAB, and rv_PA-rhlAB_I-SceI_CISR (Supplementary Table 1). Both fragments were assembled with I-SceI digested pYTSK01K_0G7 (GenBank Accession No. MT522186) to yield pYTSK10K_0G7_*rhlAB*. This vector was MauBI digested and assembled with the PCR fragment amplified from pRhon5Hi-2-eyfp using the oligonucleotides fw_CISR_eYFP and rv_eYFP_HB-R-rv (Supplementary Table 1) to yield pYTSK40K_1G7_*rhlAB*.

Assembled plasmids were isolated from yeast cultures grown in 1 mL of SD^–Ura^ medium using the innuPREP Plasmid Mini-Kit (Analytik Jena AG, Jena, Germany) with an extended lysis step (37°C, 2–5 h) in the presence of 200 U/mL *Arthrobacter luteus* Lyticase (Sigma-Aldrich Chemie GmbH, Hamburg, Germany). Yielded plasmid DNA was finally introduced into *E. coli* DH5α for amplification and isolated using the innuPREP Plasmid Mini-Kit. Competent cells of *E. coli* DH5α and *E. coli* S17-1 were prepared as previously described (Bergmans et al., 1981). The correct assembly was confirmed by sequencing (Eurofins Genomics GmbH, Ebersberg, Germany) using the “Seq” oligonucleotides shown in Supplementary Table 1. Transfer of the cloned vector to *P. putida* KT2440 (wild type) was achieved by bacterial conjugation using mating procedures. For mating, *E. coli S17-1* pYTSK40K_1G7_*rhlAB* and the *P. putida* KT2440-recipient cells were incubated for 5 h at 30°C in a droplet of LB medium on a cellulose acetate membrane on an LB agar. After the mixture was plated on *Pseudomonas*-selective LB agar, successful transposition of the integron into the attTn7 site was confirmed by colony PCR as described before (Choi et al., 2005), and the resulting strain designated *P. putida* KT2440*_*sal*_*mRL*^E^* SK40.

The construction of all other rhamnolipid producers, namely, *P. putida* KT2440 SK4, *P. putida* KT2440 Δflag SK4, *P. putida* KT2440 Δ*phaG* SK4, *P. putida* KT2440 Δpha SK4, *P. putida* KT2440 Δ*phaG* Δpha SK4, and *P. putida* KT2440 Δflag Δpha SK4, was performed by using the mini-Tn7 delivery transposon vector pSK02 as described previously (Bator et al., 2020b). Transfer of pSK02 in the wild-type and KO strains was achieved by bacterial conjugation. For mating, the recipient strain (*Pseudomonas*), helper strain *E. coli* HB101 pRK2013, transposase-leading strain *E. coli* DH5αλpir pTNS-1, and donor strain *E. coli* DH5αλpir pSK02 were used. Mating procedures were performed according to a streamlined method (Wynands et al., 2018). The rhamnolipid cassette from plasmid pSK02 was integrated into the *att*Tn7 site. Mono-rhamnolipid-producing clones were identified using cetrimide-blood agar plates [7.5% (v/v) sheep blood, Fiebig - Nährstofftechnik, Idstein - Niederauroff, Germany].

### Cultivation Conditions

#### Media

Besides the LB (lysogeny broth) medium (10 g/L tryptone, 5 g/L yeast extract, 10 g/L NaCl, Carl Roth, Karlsruhe, Germany), the mineral media enumerated in the Supplementary Material were used.

Furthermore, for the selection of *Pseudomonas*, cetrimide agar (46.7 g/L cetrimide agar powder and 10 ml/L glycerol) or LB agar with Irgasan (25 mg/L) was used. If a medium was used as a selection plate or to prevent plasmid loss, different antibiotics were added after the sterilization. Tetracycline was added in a final concentration of 20 mg/L, gentamicin was added in a final concentration of 25 mg/L and kanamycin was added in a final concentration of 50 mg/L.

#### Medium Throughput Cultivations

Microscale cultivations for the comparative analysis of different expression strains (with the inducible system) were conducted and monitored in 1.2 mL LB supplemented with 10 g/L glucose and appropriate antibiotics in sterile 48-well flower plates (MTP-48-B, m2p-labs GmbH, Aachen, Germany) using a BioLector I (30°C; 1,200 rpm, shaking diameter of 3 mm). The cultures were inoculated from precultures in LB medium with an OD_580_ (optical density) = 0.05. If appropriate, sodium salicylate was added to a final concentration of 2 mM after 3.5 h. After 24 h, samples were taken for determination of *rhlB* transcript and biosurfactant accumulation.

#### Flask Cultivations

Flask cultivation of *P. putida* KT2440 and KO strains with the strong constitutive expression system (SK4) was performed at 30°C in 500-mL shake flasks with 10% filling volume at 300 rpm or in 24-deep-well plates (System Duetz; Enzyscreen B.V., Heemstede, Netherlands) with 1 mL filling volume at 300 rpm and a shaking diameter of 50 mm. The medium was inoculated with an OD_600_ of 0.1 from a preculture in a minimal medium.

#### Physiological Characterization

Cultivation for online monitoring of the oxygen transfer rate (OTR) was carried out in an in-house built RAMOS device (Anderlei and Büchs, 2001; Anderlei et al., 2004) at a temperature of 30°C. The cells were cultured in 250-mL shake flasks with 10 mL filling volume at a shaking frequency of 250 rpm and a shaking diameter of 50 mm. The initial OD_600_ was set to 0.1. Cultivation was performed in a modified M9 minimal medium (Supplementary Material). Production of a culture supernatant for liquid–liquid extraction was carried out under the same conditions.

For variation of time of induction, 2 mM sodium salicylate from a 0.2-M stock solution in water was used to induce all cultures. Samples for offline analysis were taken from flasks run in parallel under the same conditions as the online-monitored flasks. The culture volume of one flask was sacrificed at each sampling point.

#### Fermentation

Batch fermentations for rhamnolipid production in a stirred tank reactor were conducted in a BioFlo 120 glass bioreactor (Eppendorf, Hamburg, Germany) with a working volume of 1.0 L and a nominal volume of 1.3 L. The bioreactor was equipped with a pH electrode (EasyFerm Plus PHI 225, Hamilton, Reno, NV, United States), a dissolved oxygen electrode (InPro 6800, Mettler Toledo, Columbus, OH, United States), and a Pt100 temperature sensor. The agitator shaft was equipped with two Rushton turbines (Ø 5.3 cm).

**FIGURE 2:**
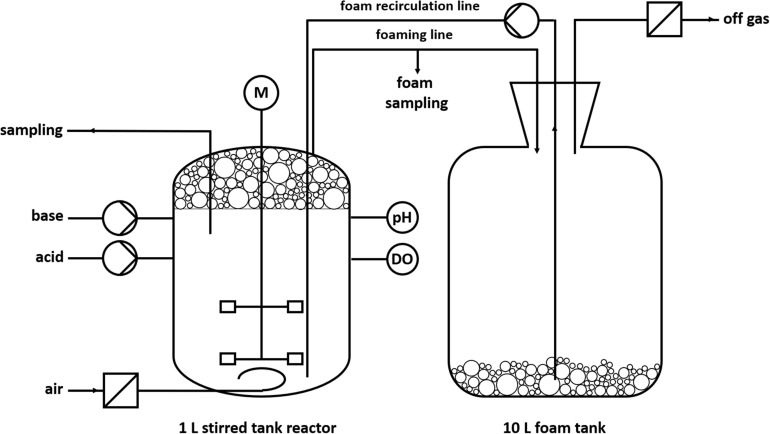
Schematic design of a batch fermentation with foam recirculation.

To handle the excessive foaming, a reactor design with foam recirculation was developed (Figure 2). The foam produced during the fermentation process is directed into a downstream foam collection bottle (10 L). The foam is pumped back into the reactor by a peristaltic pump.

The cultivations were conducted in Delft medium with standard phosphate buffer concentrations (Supplementary Material). The pH value of 7 ± 0.05 was kept constant by automatic addition of 2 M KOH and 2 M H_2_SO_4_ via peristaltic pumps. The dissolved oxygen (DO) was kept above 30% by automatic increase of the agitation speed (cascade: *t* = 0–1 h: 300–1,200 rpm; *t* = 1–10 h: 500–1,200 rpm). The aeration rate was 0.1 L/min (0.1 vvm), whereas evaporation was reduced by sparging sterile air through a sterile water bottle. The temperature was 30°C. The reactor was inoculated with cells from a preculture to an OD_600_ of 0.5. One hour after inoculation, the peristaltic pump was turned on at 220 rpm (∅_inner, hose_ = 1 cm) for foam recirculation and the stirrer speed was manually increased from 300 to 500 rpm to obtain wet foam.

### Liquid–Liquid Extraction

A pH value-dependent extraction screening of rhamnolipids from the cultivation supernatant was performed according to Biselli et al. (2020) using ethyl acetate as organic solvent (Schilling et al., 2016). Cells were removed via centrifugation. The pH values of the cultivation supernatants, in the following defined as aqueous phase, were adjusted to 1, 3, 6, and 8 with HCl or NaOH. A phase ratio (aqueous:organic) of 5 was used. The aqueous phase and organic solvent were mixed at room temperature for 30 min at 250 rpm using a vertical shaker (Hettich Lab Technologies, Tuttlingen, Germany). Subsequent phase separation was accelerated by centrifugation and was documented photographically. Both aqueous phase and organic phase were analyzed with regard to rhamnolipid concentration, samples from the aqueous phase additionally with regard to protein/peptide content. All experiments were performed in triplicates.

The performance of the liquid–liquid extraction process was evaluated, according to Biselli et al. (2020), by the reduction of rhamnolipids from the aqueous phase, using the following equation:

$$R_{\text{aq}}=\frac{m_{\text{p},\text{aq},\text{before}}}{m_{\text{p},\text{aq},\text{before}}}\cdot 100\%$$

The recovery of rhamnolipids in the organic solvent was calculated as follows:

$$R_{\text{org}}=\frac{m_{\text{p},\text{org},\text{after}}}{m_{\text{p},\text{aq},\text{before}}}\cdot 100\%.$$

Thereby, *m*_p,aq,before_ and *m*_*p,aq, after*_ are defined as the mass of rhamnolipids (P) in the aqueous phase before and after the extraction process. *m*_*p,org, after*_ is defined as mass of P in the organic phase after the extraction process.

Further, the reduction of protein/peptides from the aqueous phase during the extraction process was evaluated, as follows:

(1) $$L=\frac{m_{\text{prot},\text{aq},\text{before}}-\,m_{\text{prot},\text{aq},\text{after}}}{m_{\text{prot},\text{aq},\text{before}}}\cdot 100\%.$$

Since Biselli et al. (2020) showed that liquid–liquid extraction of rhamnolipids from the cultivation supernatant based on the LB medium using ethyl acetate is highly selective with purities of rhamnolipids in ethyl acetate close to 100 w%, purities will not be investigated at this point.

### Environmental Assessment of Rhamnolipid Production

An LCA was carried out for a technical scale with an operation volume of 112.5 L (total volume 150 L) in the fermentation stage, in line with ISO standards (ISO14040, 2006; ISO14044, 2006).

The LCA was performed with the software GaBi 9.2, the associated GaBi database, and the ecoinvent database 3.5 (ecoinvent-DB, 2020; GaBi-DB, 2020; GaBi-S, 2020). Addressees of the generated results are mainly the involved scientists. The LCA is applied to the product chains of rhamnolipid production with two different substrates from the sugar industry. Process chain RL_I operates with molasses, while RL_II operates with hydrolyzed sugar beet pulp.

#### Goal and Scope Definition for LCA

The goal of the study was to identify impact-, energy-, or material-intensive process stages of the described system. Furthermore, it can be illustrated which processing route is more advantageous in relation to the efficiency of substrates. The correlation between LCA and process design is principally applicable for an iterative but continuous improvement of the process chains.

The assessed cradle-to-gate system, as described below, includes the basic process modules of the process chains. Additional equipment, like it is used in an industrial scale (e.g., pumps and storage tanks), is not considered in the present study. The described components are the same for both abovementioned process variants and include the production and operation of each component. Furthermore, the production of input flows like water, substrate, and auxiliary materials (e.g., solvents and minimal medium) is covered. The geographical and time-related system boundaries are related to the sugar beet production area in North Rhine-Westphalia (Germany) at present. As the functional unit, the production of 1 kg of rhamnolipids is defined. The used data (e.g., fermentation yields, conversion rates, aeration rates, and purge share) was provided by further project partners and originate from studies in the lab scale. Additional data was taken from databases (background processes; e.g., GaBi-DB, 2020), literature (e.g., Knoll, 2008; Spoerri and Thomas, 2014), statistical data (e.g., WVZ, 2020), or own calculations (e.g., tank design). For the life-cycle impact assessment (LCIA), the recommended methods of ILCD (Pennington et al., 2010; Wolf et al., 2012) were applied and any normalization carried out was performed in line with PEF Pilot 1.09 [(Benini et al., 2014), person equivalents (PE) related to EU-27].

The environmental impact is expressed by 16 impact categories enumerated in the Supplementary Table 2.

The normalization follows the conversion of results with specific impact category units (e.g., CO_2_ equivalents to PE by the application of specific characterization factors). One PE represents the summarized emissions of an average yearly European citizen generated by consumption. Data for the supply of molasses or sugar beet pulp were taken from an existing LCA for sugar beet production and processing (Spoerri and Thomas, 2014) and allocated by the economic value share of the substrate. An allocation ensures that environmental impacts from upstream processes are only added proportionately, as the used substrates are only one of several upstream chain products.

#### Process Development

Processes of rhamnolipid production were developed based on the results generated by laboratory-scale studies. The resulting process chains of RL_I and RL_II were not examined in terms of a connected system, so that the design was to a limited degree completed by additional, but secondary, components (e.g., drying). The dimensioning of specific components was oriented to the size of the fermentation. Required components for large-scale implementation (e.g., pumps) were not included in the technical scale, but utilization routes for residues or by-product flows were contained in the design. Depending on the used substrate, different upstream process modules were used, due to the fact that sugar beet pulp needs some preprocessing steps (disintegration and hydrolyzation) before usage. The following stages of fermentation and downstream processing are qualitatively the same in both cases, whereby the differing substrate-depending parameters (e.g., density, sugar content, and dilution rate) were considered by calculations and the resulting consequences for dimensioning or throughput, for instance (see “Life-Cycle Inventory” section). While one of the developed process chains is illustrated and explained in the result section, the second can be found in the Supplementary Figure 2.

#### Life-Cycle Inventory

Table 3 illustrates the incoming and outcoming masses as well as further parameters of the production process chains RL_I and RL_II in a 150-L scale. In consideration of specific sugar contents, yield coefficients, and conversion rates, the necessary amount of substrate (theoretical demand) differs by factor 1.4 (1 unit sugar beet pulp = 1.4 units molasses). The assessed 150-L fermentation system (112.5 L operation volume) in combination with the specific yield of 0.1 kg_Product_/kg_Gluose_ can produce a 0.211 kg (rhamnolipids and molasses) and 0.316 kg (rhamnolipids and sugar beet pulp) product per volume change of the fermentation, which results in a number of three batches in minimum to achieve the intended 1 kg of product. Further microorganism-specific studies under laboratory conditions have shown yields with coefficients up to 0.2 kg_Product_/kg_Gluose_ (without optimization measures), which would cut down the results of PE massively. For the fermentation stage, the dilution of substrate is approximately 50% water for molasses-driven processes and 60% for sugar beet pulp-driven processes. Additional inputs to the fermentation process are the minimal medium (13.2 g_Medium_/kg_Fermentation broth_) and inoculum [0.5% (v/v)].

**TABLE 3:** Life-cycle inventory of rhamnolipid production-process chains RL_I and RL_II for the use of a 150-L batch-fermentation process.

**Basic parameter**	**Unit**	**RL_I**	**RL_II**
Product	[–]	Rhamnolipid	Rhamnolipid
Substrate	[–]	Molasses	Sugar beet pulp (hydrolyzed)
Content usable sugar	[kg_Glucose_/kg_Substrate_]	0.47	0.67
Yield coefficient	[kg_Product_/kg_Glucose_]	0.1	0.1
	[kg_Product_/kg_Substrate_]	0.05	0.07
Conversion rate	[%]	10	10
**Inputs**			
Substrate	[kg_Substrate_/kg_Product_]	212.8	150.2
Substrate per 150-L fermentation	[kg_Substrate/Fermentation_]	76.4	39.6
Water per 150-L fermentation	[kg_Water/Fermentation_]	55.1	66.1
Minimal medium per 150-L fermentation	[kg_Minimal medium/Fermentation_]	1.46	1.46
Inoculum per 150-L fermentation	[kg_Inoculum/Fermentation_]	0.66	0.66
Total operation volume	[L_Fermentation content_]	112.5	112.5
Specific aeration rate	[L_Gas_/L_Fermentation broth_/min]	0.1	0.1
Precipitation agent	[kg]	120.2	91.7
Recycling rate	[%]	80	80
Extraction agent	[kg]	18.0	17.0
Recycling rate	[%]	80	80
Acidification agent	[kg]	0.012	0.011
**Intermediate products**			
Fermentation broth	[kg]	133.6	107.8
Theoretical contained RL	[kg_RL_]	0.359	0.240
Product losses	[kg]	0.043	0.029
**Output**			
Product	[kg]	0.316	0.211
Solid residues	[kg]	13.3	18.5
Liquid residues	[kg]	129.7	102.2

*RL, rhamnolipid.*

Further process modules require different—substance flow mass depending—amounts of precipitation agent (acetone) and extraction agent (ethyl acetate), which are recycled by a defined default share of 80%, and acidification agents. The outcoming theoretical product amounts of 0.359 kg (molasses) and 0.240 kg (sugar beet pulp) are contained in a fermentation broth output of 133.6 kg for the molasses-driven process and 107.8 kg for the sugar beet pulp-driven process. The product losses throughout the entire process (e.g., during extraction) can be quantified with ca. 12% for both production processes.

### Analytics

#### Optical Density

For determination of the optical density, a spectrophotometer (Genesys 20, Thermo Fisher Scientific, Waltham, MA, United States) was used for the experiments to determine the optimal induction point, while for the experiments regarding minimal medium and chassis optimization an Ultrospec 10 Cell Density Meter (Biochrom GmbH, Berlin, Germany) was used. The optical density was measured in 1-cm cuvettes at a wavelength of 600 nm. Prior to measurement, the sample was appropriately diluted using 0.9% NaCl. The measurement was carried out in duplicate for each sample.

For the correlation of OD_600_ to cell dry weight (CDW), the factor of 0.3 was determined for the Ultrospec spectrophotometer.

#### pH Value

The pH value was determined with an InLab Easy pH electrode (Mettler Toledo, Germany) and a CyberScan pH 510 m (Eutech Instruments, Thermo Fisher Scientific, Waltham, MA, United States). Appropriate buffers were used for calibration.

#### Carbon Source Quantification

The glucose concentration in the experiments to determine the optimal induction point was determined by HPLC (Dionex Ultimate 3000, Thermo Fisher Scientific, Waltham, MA, United States) using a refractive index detector (RefractoMax 521, Thermo Fisher Scientific, Waltham, MA, United States). An organic acid resin column (250 mm × 8 mm, CS-Chromatographie Service, Langerwehe, Germany) was used for separation at a temperature of 40°C. 1 mM sulfuric acid was used as a mobile phase. A flow rate of 0.8 mL/min was used.

For the experiments regarding minimal medium and chassis optimization, glucose, gluconate, and ketogluconate were quantified by using a Beckmann Coulter System Gold HPLC with a UV detector 166 (Beckmann Coulter, Brea, CA, United States) at 210 nm and a refractory index detector RI2300 (Knauer GmbH, Berlin, Germany). For separation, a Metab-AAC 300 mm × 7.8 mm column (particle size: 10 μm, ISERA GmbH, Düren, Germany) was used. Elution was performed with 5 mM H_2_SO_4_ at a flow rate of 0.5 mL/min at 40°C.

#### Qualitative Detection of Rhamnolipids via Hemolysis Assay

Blood agar plates for hemolysis assays were prepared with 40 g/L blood agar basis (Carl Roth, Karlsruhe, Germany) in water. After autoclaving and cooldown to below 50°C, 10% (v/v) defibrinated sheep blood (Fiebig Nährstofftechnik GbR, Idstein, Germany) and, if applicable, antibiotics and sodium salicylate (5 mM) were added. Bacteria were applied to the solid medium using a 96-pin replicator. After overnight growth at 30°C, the plates were stored at 4°C. Lysis halo formation was documented daily using a CAMAG TLC Visualizer 2 (Camag AG & Co. GmbH, Muttenz, Switzerland).

#### Quantification of Rhamnolipids

For sample preparation, the cell-free culture broth was mixed 1:1 with acetonitrile and stored at 4°C overnight. Subsequently, the mixture was centrifuged at 11,000 × g for 2 min. All samples were filtered with Phenex-RC syringe filters (0.2 μm, ∅ 4 mm, Phenomenex, Torrance, CA, United States). Reversed-phase chromatography couple to Corona Charged Aerosol Detection (CAD) was performed to quantify rhamnolipid and HAA concentrations based on a previously developed method (Behrens et al., 2016; Tiso et al., 2016) using an Ultimate 3000 with a Corona Veo Charged Aerosol Detector (Thermo Fisher Scientific, Waltham, MA, United States). For separation, a NUCLEODUR C18 Gravity 150 mm × 4.6 mm column (particle size: 3 mm, Macherey-Nagel GmbH & Co. KG, Düren, Germany) was used. The flow rate was set to 1 mL/min, and the column oven temperature was set to 40°C. Acetonitrile (A) and 0.2% (v/v) formic acid in ultra-pure water (B) were used as running buffers. The method started with a ratio of 70% buffer A:30% buffer B and a linear gradient was applied to reach a ratio of 80%:20% in 8 min. The acetonitrile fraction was increased linearly from 80 to 100% between 9 min and 10 min and decreased linearly to 70% between 11 min and 12.5 min. The measurement was stopped after 15 min.

For the purpose of this paper, the term “rhamnolipids” refers to the resulting natural product mixture containing mono-rhamnolipids and a small fraction of the aglycon HAA.

#### Transcript Levels

Total RNA was isolated from harvested cells equivalent to an OD_580_ = 2 in 1 mL using a NucleoSpin RNA Kit (Macherey-Nagel GmbH & Co. KG, Düren, Germany) according the manufacturer’s instructions with an additional DNA digestion step (RNase-Free DNase Set, Qiagen GmbH, Hilden, Germany) on the spin filter. After elution, potentially remaining DNA was eliminated using the DNA-free DNA Removal Kit (Thermo Fisher Scientific GmbH, Waltham, MA, United States). The extraction yielded 17.4 μg ± 1.6 for strain pPS05, 26.5 μg ± 2.3 for strain SK4, and 33.728 μg ± 8.5 for strain SK40 (means and standard deviations of biological triplicates). The RT-qPCR (reverse transcription and quantitative PCR) was performed with Power SYBR Green RNA-to-CT 1-Step Kits (Thermo Fisher Scientific GmbH, Waltham, MA, United States) according to the manufacturer’s instruction and run on an Applied Biosystems 7900HT Fast Real-Time PCR System (Thermo Fisher Scientific GmbH, Waltham, MA, United States). The runs were analyzed using the software SDSv2.3 (Thermo Fisher Scientific GmbH, Waltham, MA, United States). Amplicons (136 bp) of *rhlB* were generated using the primers PA-rhlB_fw-RT and PA-rhlB_rv-RT (Supplementary Material and Table 3). The primers were designed using the Primer3 Web tool (Kõressaar et al., 2018). To each reaction, 50 ng of the total RNA preparation was added. For calibration, serial dilution of vector pYTSK40K_1G7_*rhlAB* was used as qPCR template in technical triplicates, allowing for calculation of *rhlB* transcript copy numbers (Supplementary Figure 1). Copy numbers per OD were calculated based on the initially extracted total RNA from cells equivalent to OD_580_ = 2 in 1 mL. For statistical analysis of the differences between the expression systems, an unpaired student’s *t*-test was applied. We routinely analyzed melting curves to ensure primer specificity (Bustin et al., 2009) (Supplementary Figure 1). The qPCR results were additionally verified by using reaction mixtures containing H_2_O instead of RNA and reaction mixtures without reverse transcriptase as negative controls for each biological sample.

#### BCA Protein Assay

Protein/peptide content of aqueous samples in liquid–liquid extraction was determined by BCA Protein Assay (Thermo Fisher Scientific, Waltham, MA, United States) according to Biselli et al. (2020).

## Results

**FIGURE 3:**
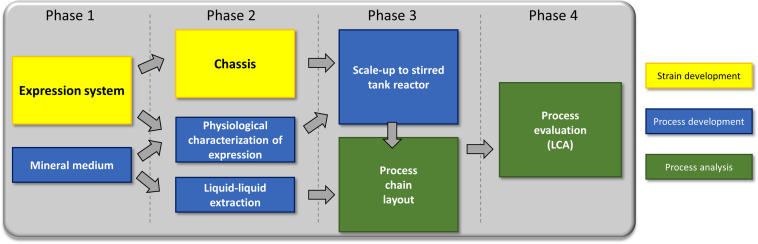
The sequence in which the experiments have been carried out in this study.

To address the challenges associated with rhamnolipid production on all levels, strain engineering, process development, and environmental impact were simultaneously considered. Necessary changes were communicated back to the preceding process stage (Figure 3).

Phase 1: We established a strategy for rhamnolipid production in a non-pathogenic strain and a suitable medium sustaining growth and high production. Feedback from the design of downstream processing led to discarding media with high protein/peptide contents (LB medium). Phase 2: Based on these prerequisites, we now streamlined the strain, evaluated the inducible expression system, and designed a downstream process. Phase 3: The gained insights were subsequently used to establish a lab-scale bioreactor fermentation and to design a basic process. Phase 4: Finally, all knowledge was used to do a process evaluation (LCA).

### Inducible Expression of Chromosomally Integrated Rhamnolipid Synthesis Genes Entails Genetic Stability

Plasmids with strong synthetic promoters have been commonly used for the expression of the genes for rhamnolipid biosynthesis in studies on rhamnolipid production with *P. putida* (Loeschcke and Thies, 2020). Contradictorily, chromosomal integration of expression modules is regarded in general as beneficial for stable production strain development circumventing the need for supplementation of antibiotics (Li et al., 2019). We compared here advanced expression cassettes for the stable integration of the *P. aeruginosa* PAO1 *rhlAB* operon encoding mono-rhamnolipid biosynthesis into the *att*Tn7 site of the genome with the state-of-the-art plasmid-based system (Beuker et al., 2016a; Tiso et al., 2016). Besides strain SK4 harboring a cassette with a strong constitutive promoter that was recently established (P_14ffg_) (Köbbing et al., 2020) and already used for mono-rhamnolipid production in *P. putida* (Bator et al., 2020b), we evaluated also in strain SK40 an inducible expression system based on NagR/P_nagAa_, which proved very promising in previous studies for the production of bioactive secondary metabolites in *P. putida* (Schmitz et al., 2015; Bator et al., 2020b).

**FIGURE 4:**
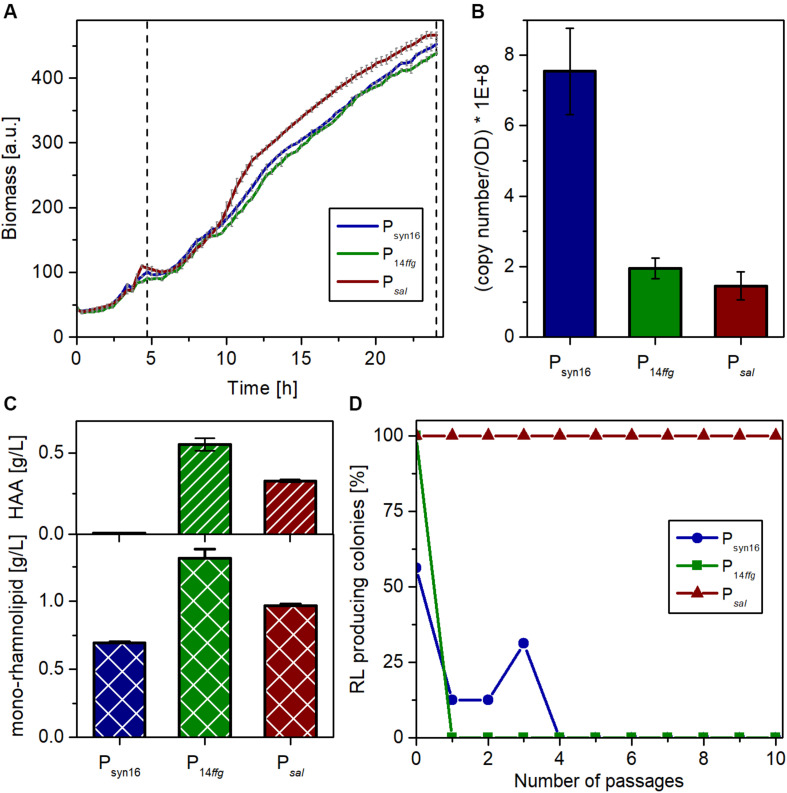
Characterization of *P. putida* carrying genome-integrated *rhlAB* expression cassettes in comparison with *P. putida* KT2440 pPS05. **(A)** Growth of *P. putida* KT2440 containing plasmid pPS05 (P_syn16_), or integrons P_14ffg_*rhlAB* (strain SK4) and P_*nagAa*_*rhlAB* (strain SK40), respectively, in flower plates with LB medium supplemented with glucose. The shown curves represent mean values of three independent cultures with standard deviation. The dashed vertical line marks the induction time (for P_nagAa_*rhlAB*) and sampling. **(B)** Copy number of the *rhlB* transcript after 24 h per cell number equivalent to an OD_580_ = 1 in 1 mL as determined by RT-qPCR. RNA was prepared from three biological replicates. The shown values represent mean values of the three replicates with standard deviation. **(C)** Titers of HAA (top) and mono-rhamnolipid (bottom) in the supernatants of the expression strains after 24 h as determined by HPLC. The shown titers represent mean values of three biological replicates with standard deviation. **(D)** Stability of rhamnolipid production of the different strains over many generations, during passaging on LB agar plates under selection pressure. After each passage, the grown colonies were transferred to blood agar with 5 mM salicylate if applicable and incubated overnight at 30°C. Plotted is the remaining percentage of producing colonies among the total number of colonies (P_syn16_ and P_14ffg_: 16 colonies, P_*sal*_: 24 colonies) after each passage on LB.

Three independent microscale cultivations of each strain in the LB medium with 10 g/L glucose were conducted in parallel for 24 h. 3.5 h after inoculation, the cultivation was shortly interrupted to supplement the SK40 cultures with 2 mM of salicylate for the induction of the NagR-dependent expression system. Biomass formation was continuously monitored via scattered light. While the growth behavior of all three strains was similar, the inducible strain reached slightly higher growth rates and final cell densities (Figure 4A). After 24 h, the expression strength of the complete biosynthetic operon *rhlAB* was determined by RT-qPCR as transcript copies of *rhlB*, the second gene of this transcriptional unit. The strain with plasmid-based expression (pPS05) showed stronger *rhlB* transcript accumulation than the other two strains, which may be connected to a gene dosage effect evoked by the higher copy number of plasmids (Figure 4B). P_14ffg_-driven expression appeared to be stronger than the inducible system, which was expected considering the strong activity of the synthetic promoter. However, this difference was not significant. The supernatants of all cultures were analyzed with regard to the achieved rhamnolipid titers (this includes besides the mono-rhamnolipid also the aglycon HAA). In accord with the transcript analysis, the ffg expression cassette in strain SK4 led to the accumulation of the highest concentration of rhamnolipids among the three systems tested with nearly 1.9 g/L (Figure 4C). The salicylate inducible expression (SK40) led to titers around 1.3 g/L. Remarkably, the plasmid-based expression yielded the lowest titer under the chosen conditions.

One general advantage of genome-integrated expression cassettes over the utilization of plasmids is the stability of the resulting production strain. Hence, the three strains were compared regarding the maintenance of their capability to produce rhamnolipids. To this end, we performed a passaging experiment on agar plates and determined the percentage of rhamnolipid-producing colonies after each passage. Preculture from cryostocks was used to inoculate in total 56 cultures. After growth overnight, samples were spotted onto blood agar plates (with salicylate in case of NagR/P_nagAa_ strains) to determine the situation at the start [number of passages (P) = 0], and onto LB-agar plates. After overnight incubation, the formed colonies were transferred from the LB plates onto (i) again blood agar plates (*P* = 1) and (ii) fresh LB agar. This was repeated nine times. Selection pressure by antibiotics was maintained during the whole experiment to avoid mere plasmid or integron loss. The results revealed that the capability for rhamnolipid production is more stable using a genome-integrative system, while the plasmid bearing strain showed heterogeneity already at *P* = 0 (Figure 4D). *P*_14ffg_ strains also lost rapidly the ability to produce rhamnolipids during the passaging. The apparent regaining of production by three clones at (*P* = 3) is an artifact of the completely qualitative nature of the experiment. In contrast, the inducible strain, which was passaged under non-inducing conditions, maintained its ability for rhamnolipid production. Conclusively, constitutive expression of *rhlAB* genes appeared to exhibit strong selection pressure that led to loss of function, although this was not directly visible from the growth curves. Hence, strain stability was massively increased compared to the state-of-art-system by genome integration of the expression cassette and by inducible expression.

### Efficient Rhamnolipid Production Using Minimal Media

The usage of the LB medium complemented with glucose seems to be highly suited for the synthesis of rhamnolipids, as medium components are used for cell growth, while the glucose is supplied to the rhamnolipid synthesis pathway (Wittgens et al., 2011). This medium is, therefore, commonly used in fundamental research with microbiological focus. However, for industrial-scale processes, complex media are not suited. Furthermore, previous work within our consortium has demonstrated that the high protein/peptide content therein is detrimental for downstream processing (Biselli et al., 2020).

**FIGURE 5:**
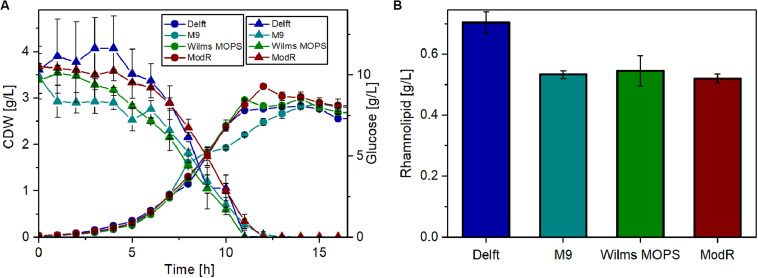
Rhamnolipid production of *P. putida* KT2440 SK4 with different minimal media. **(A)** Growth of *P. putida* KT2440 SK4 on four different minimal media and the course of the glucose concentration. **(B)** Rhamnolipid titers after 14 h of cultivation. Cultivation conditions: 500-mL shake flasks with 50 mL filling volume, mineral media with 10 g/L glucose at a shaking frequency of 300 rpm with a shaking diameter of 50 mm, and an initial optical density of 0.1. The error bars are based on two biological duplicates.

Thus, four different minimal media were tested for their suitability for rhamnolipid production using glucose as the sole carbon source. For these experiments, the strain that was previously observed (Figure 4) to have the highest metabolic capacity was used (*P. putida* KT2440 SK4). The results (Figure 5) show that growth on all tested media is similar. After around 13 h, the cell dry weight (CDW) reached approximately 3 g/L. Also, glucose depletion was comparable, as after 12 h glucose was completely consumed in all cases. The highest rhamnolipid concentrations reached during the cultivations (Figure 5B) indicated that the Delft medium is best suited for rhamnolipid synthesis using recombinant *P. putida* KT2440 SK4 (700 mg/L). The yield observed was 0.06 g_RL_/g_Glc_, which amounts to 14% of the theoretically possible yield. Compared to the other media, the Delft medium allowed an increase in rhamnolipid titers of about 30%.

### Chassis Optimization by Decreasing the Metabolic and Energetic Burden of the Cell

Up to now, the metabolic burden opposed on the cells by introduction of the non-native rhamnolipid synthesis operon has been evaluated. Additionally, here, it is attempted to lower the intrinsic metabolic burden by deleting high-energy or resource-demanding side activities. Consequently, the expression with the highest metabolic demand was chosen (the constitutive promoter P_14ffg_). The first target was the flagellar machinery (flag), which has been shown to consume a considerable amount of metabolic currency. Furthermore, the relevant genes amount to about 1% of the genome (Martinez-Garcia et al., 2014). Removing this structure might free up resources, which can be used to synthesize secondary metabolites like rhamnolipids. The second target was the pathway for polyhydroxyalkanoate (PHA) synthesis. It has previously been shown that the precursor *R*-3-hydroxyacyl-CoA is predominantly involved in rhamnolipid and PHA production in *P. aeruginosa* (Abdel-Mawgoud et al., 2014). Nevertheless, it is believed that *R*-3-hydroxyacyl-ACP generated by fatty acid *de novo* synthesis is also used for rhamnolipid and PHA production. Although the involvement of *de novo* synthesis or β-oxidation for rhamnolipid production in *P. putida* is not investigated, the potential competition of the precursor needed for rhamnolipid synthesis was addressed. As shown by Rehm et al. (2001), the gene *phaG* is present in *P. putida*, which encodes PhaG, a 3-hydroxyacyl-acyl carrier protein-coenzyme A transferase. PhaG transfers a CoA to the hydroxy fatty acid, releasing the ACP (Rehm et al., 1998). Subsequently, the PHA polymerases (PhaC1 and PhaC2) start forming the polymer. The genes *phaC1* and *phaC2* are clustered together with the gene for the PHA depolymerase *phaZ*, *phaD* (a transcription regulator), *phaF*, and *phaI* (PHA granule-associated proteins, phasins). The gene *phaG* is in a different locus. Abolishing PHA synthesis might relieve the metabolic network and direct the metabolic flow into rhamnolipid synthesis. In this study, *phaG*, and the complete PHA operon (*phaC1ZC2DFI*) are knocked out individually and in combination.

**FIGURE 6:**
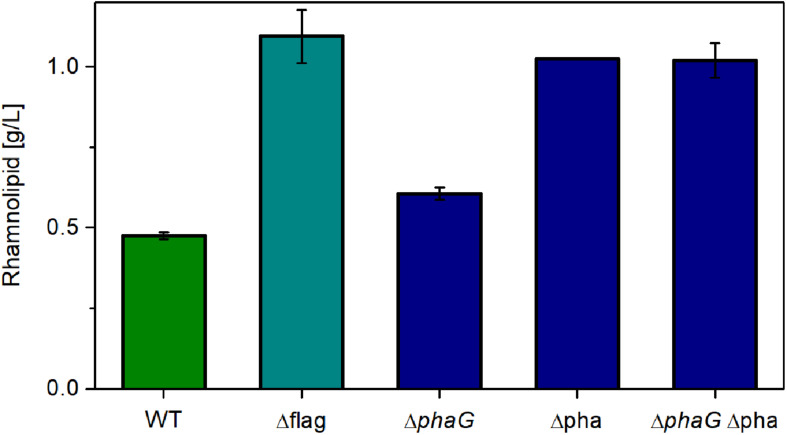
Chassis optimization. Rhamnolipid production of different knockout mutants using the constitutive expression system. Cultivation conditions: 500-mL shake flasks with 50 mL filling volume, Delft medium with 10 g/L glucose at a shaking frequency of 300 rpm with a shaking diameter of 50 mm and an initial optical density of 0.1. Samples were taken after 24 h. The error bars are based on biological duplicates.

As expected, relieving *P. putida* of the metabolic burden to operate the flagellar machinery led to an increase in rhamnolipid synthesis (Figure 6, Δflag). As control, we used the knockout free rhamnolipid producer SK4, which produced 0.47 g/L rhamnolipids. Compared to this strain, the flagellum knockout strain achieved a 130% higher titer. A similar increase was observed when deleting genes from the PHA-synthesis pathway. Knocking out *phaG* led to an increase of 27% in the rhamnolipid concentration while the knockout of the complete PHA operon effectuated a doubling in rhamnolipid titers (115% increase). The increase of the rhamnolipid concentrations caused by the deletion of *phaG* indeed in all likelihood indicates an involvement of fatty acid *de novo* synthesis for the supply of the precursor for PHA and rhamnolipid production. Knocking out all genes involved in PHA synthesis (*phaG* and *phaC1ZC2DFI*) did not increase rhamnolipid titers further. The best-producing strain (Δflag) featured a carbon yield of 0.10 g_RL_/g_Glc_, which amounts to 22% of the theoretically possible yield. This poses a significant increase compared to the 14% achieved with the wild-type strain. The lower rhamnolipid titer in comparison to Figure 5 might be caused by rhamnolipid degradation after the carbon source was depleted (sample time was 24 h). Although we usually do not see degradation of rhamnolipids, this might have happened here.

### Early Induction Leads to Higher Productivity

**FIGURE 7:**
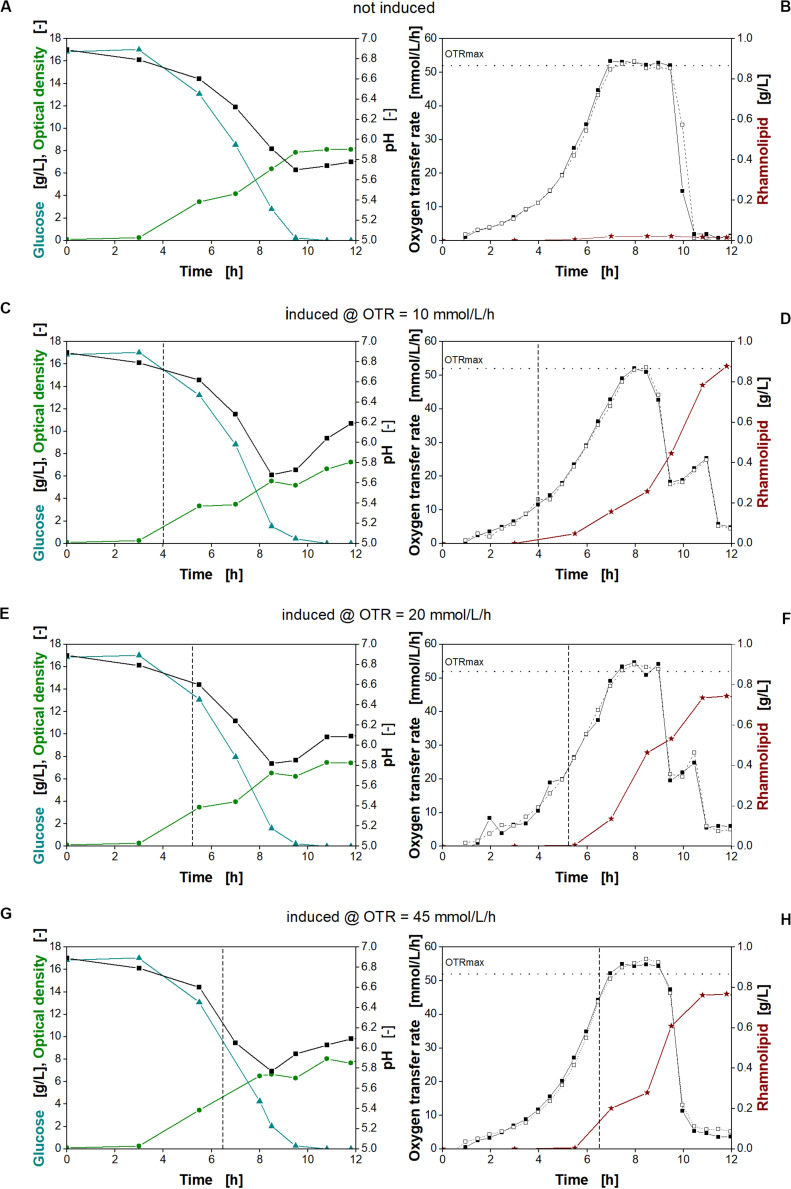
Physiological studies of *P. putida* KT2440 SK40 for variation of time of the time point of induction with salicylate. Detailed physiological data for glucose (triangles), pH (squares), and optical density (stars) **(A,C,E,G)** as well as oxygen transfer rate (OTR) (closed and open squares) and surfactant concentration (stars) **(B,D,F,H)** at different times of induction. The dashed vertical line marks the induction time point. The dotted horizontal line marks the maximum oxygen transfer capacity (OTRmax) according to Meier et al. (2016). Induction with 2 mM salicylate after 4 h **(C,D)**, 5.3 h **(E,F)**, and 6.5 h **(G,H)** and non-induced culture **(A,B)**. Cultivation conditions: 250-mL shake flasks with 10 mL filling volume, M9 minimal medium (17 g/L glucose, 0.1 M MOPS buffer, pH 7) at a shaking frequency of 250 rpm with a shaking diameter of 50 mm and an initial optical density of 0.1.

Previously, the expression levels and thus the synthetic metabolic burden (Figure 4) and production in a minimal medium (Figure 3) have been assessed separately. By revising the induction time point using the inducible P_Sal_ promoter in the minimal medium, we might be able to modulate the expression level (and thus metabolic stress on the cell) further. Consequently, the influence of different induction time points on strain *P. putida* KT2440 SK40 was investigated by online monitoring of the oxygen transfer rate (OTR) for detailed physiological and metabolic characterization. Two mM salicylate was added at different cell densities to investigate the impact of different induction time points on rhamnolipid formation. The data are presented in Figure 7.

An exponential increase in the OTR was observed for all cultures (Figures 7B,D,F,H, squares). This increase continues after induction, indicating no strong metabolic burden. Typically, a strong metabolic burden is reflected by visible changes in the OTR after induction (Rahmen et al., 2015a,b; Ihling et al., 2018; Mühlmann et al., 2018). The maximum oxygen transfer capacity (OTRmax) under the chosen cultivation conditions was determined to be 52 mmol/L/h according to Meier et al. (2016). Consequently, all cultures reach an oxygen limitation indicated by a constant OTR (Figures 7B,D,F,H). After depletion of glucose, a smaller peak in the OTR curves at 10 and 20 mmol/L/h (Figures 7D,F, squares) indicates diauxic gluconate and ketogluconate consumption (Supplementary Figures 3B,C, respectively). For the non-induced culture and the culture induced at an OTR of 45 mmol/L/h (Figures 7B,H, respectively), this second peak is not visible, because the majority of gluconate and ketogluconate was already metabolized during the oxygen limitation (Supplementary Figures 3A,D, respectively). In addition, the measurement values of the OTR were recorded 30 min apart from each other, so that the consumption of the residual remaining gluconate and ketogluconate is not clearly visible. The diauxic growth behavior was also reflected by the changing increase of the biomass concentration seen previously (Figure 4A). Since gluconate is metabolized, no inhibition by pH is observed (Scheidle et al., 2011). At the end of the cultivation, all cultures reach a comparable optical density (Figures 7A,C,E,G, green circles).

As expected, no rhamnolipids were produced, if the culture was not induced (Figure 7B, stars). Induction at increasing OTR values, corresponding to increasing biomass concentrations, reveals that an early induction at an OTR of around 10 mmol/L/h was favorable for higher rhamnolipid titers. Consequently, the rhamnolipid titers from early-induced cultures exceeded the titers obtained at the other conditions by 18%. These observations fit with the high titers observed with constitutive promoters. As no metabolic burden was observed, induction could be performed right from the beginning. The main advantage of the inducible system may be to conduct seed fermentations under non-inducing conditions.

The data also clearly show that, despite being oxygen limited during cultivation, all induced cultures continue the production of rhamnolipids. However, the different efficiencies show that rhamnolipid synthesis seems thus to be sensitive to small changes in the cultivation conditions.

### The Usage of Minimal Medium Entails Advantages in Liquid–Liquid Extraction

To design an overall efficient production process, efficient separation and purification are as important as the development and utilization of efficient production strains and fermentation conditions. A previous study evaluated the pH value and phase ratio dependent liquid–liquid extraction of rhamnolipids from cultivation supernatants produced based on LB medium using ethyl acetate as organic solvent (Biselli et al., 2020). It was shown that proteins/peptides in the cultivation supernatant accumulate in interphases in the liquid–liquid extraction process at acidic pH values. Further, the observed interphase formation was coupled with a reduced recovery of rhamnolipids in ethyl acetate. Here, we evaluated how far interphase formation and the recovery of rhamnolipids in ethyl acetate is influenced by the use of cultivation supernatants produced based on minimal medium instead of LB medium.

Table 4 depicts the results of the pH value-dependent extraction screening using cultivation supernatant produced based on minimal medium. During the liquid–liquid extraction, a pH value-dependent interphase formation was observed (Table 4). Thereby, strong interphase formation occurred for pH values of 1 and 8, while moderate interphase formation for a pH value of 6, and only minor interphase formation for a pH value of 3 was observed. For acidic pH values (1, 3, 6), a rhamnolipid reduction from the aqueous phase above 98 w% was observed. At a pH value of 8, a rhamnolipid reduction from the aqueous phase of 74.4 w% was observed. However, all samples with interphase formation showed strongly reduced recoveries of rhamnolipids in the organic phase (23.3–64.9 w%). At a pH value of 8, no clear organic phase could be separated. Accumulation of proteins/peptides in the interphase appears probable because of the qualitative degree of interphase formation, which correlates with the amount of protein/peptide reduction from the aqueous phase. Further, the experiment revealed a relation between interphase formation and reduced recovery of rhamnolipids in the organic solvent, which is in line with previous reports. An accumulation of rhamnolipids in interphases is assumed (Schilling et al., 2016; Biselli et al., 2020).

**TABLE 4:** Results of the liquid–liquid extraction of rhamnolipids from cultivation supernatant produced based on minimal medium.

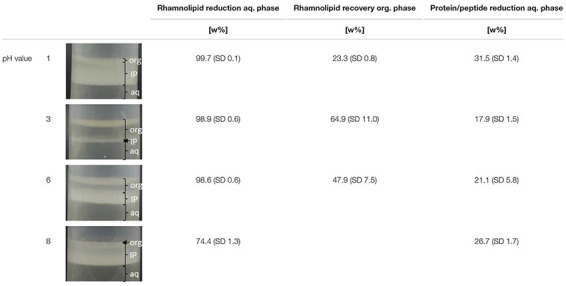

*The presented excerpts of photos show the pH value-dependent interphase formation. Standard deviation is based on triplicates (Org, organic phase; IP, interphase; Aq, aqueous phase; SD, standard deviation).*

### Foam Recycling in a Bioreactor Setup Allows Efficient Rhamnolipid Production

From the previous experiments, the following conclusions can be drawn: The maximal burden for the cells with high expression levels (=synthetic metabolic burden high) in the wild-type background (=intrinsic burden normal) led to rhamnolipid titers of 0.5–0.7 g/L (minimal medium experiments). Maintaining the high intrinsic burden (wild-type background) but decreasing the synthetic burden (adjusting the induction time point) led to higher titers (0.9 g/L). By the reversed experiment with high expression levels and lowered intrinsic metabolic burden (by metabolic engineering), the so far highest titers could be achieved (1.1 g/L). For the scale-up experiment, *P. putida* KT2440 Δflag SK4 was chosen.

For the first scale-up of the rhamnolipid production process, a batch fermentation procedure was established in a 1-L bioreactor. Due to the surface-active properties of the product, a special reactor design is required, since an aerated biosurfactant fermentation process leads to strong foam formation. A very simple and robust bioreactor setup with continuous foam recycling was developed for this purpose. Using this system, it was possible to control the foaming without the usage of antifoaming agents or mechanical foam breakers, which can have a significant negative impact on the performance and on the costs of the process. Especially the purification of the product becomes challenging when antifoaming agents are used, as the product has to be isolated from an emulsion.

The batch fermentations were conducted as biological duplicates with *P. putida* KT2440 Δflag SK4 using the Delft medium supplemented with 10 g/L glucose as sole carbon source. The foam produced during fermentation was collected in a 10 L foam tank and left undisturbed to collapse. The collapsed foam was continuously pumped back into the reactor, which nearly completely prevented the loss of biomass and product. This avoided a reduction in productivity over time due to loss of biomass in the foam.

**FIGURE 8:**
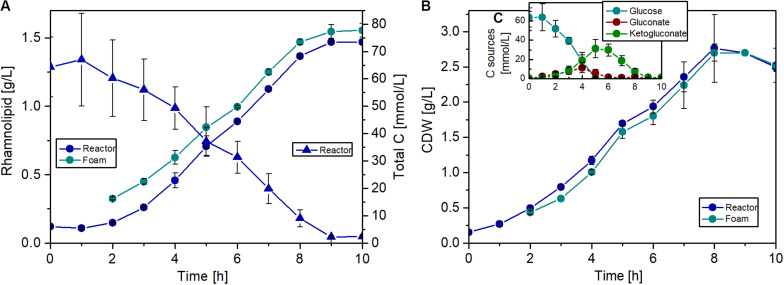
Fermentation of *P. putida* KT2440 Δflag SK4 for rhamnolipid production. **(A)** Courses of rhamnolipid (circles) and total carbon (triangles) concentrations (including glucose, gluconate, and 2-ketogluconate) in the fermenter (blue) and in the foam (cyan). **(B)** Courses of cell growth in CDW in the fermenter (blue) and in the foam (cyan). **(C)** Concentrations of the single carbons: glucose (cyan), gluconate (red), and ketogluconate (green). Cultivation conditions: 1.3 L bioreactor with 1 L working volume, Delft medium with 10 g/L glucose, an aeration rate of 0.1 L/min (0.1 vvm), and an initial optical density of 0.5. The error bars are based on biological duplicates.

The fermentations were carried out over 10 h, and a maximum biomass concentration of 2.8 g/L with a maximal growth rate of 0.56 1/h was achieved. Remarkably, during the whole fermentation, the biomass concentration in the foam phase was comparable to the biomass concentration in the reactor, if not slightly lower (Figure 8B). This might be a positive effect of the knockout, since usually the biomass is accumulating in the foam (Beuker et al., 2016b). The break in the growth and production curves after 5 h (Figures 8A,B) can be explained by glucose depletion (Figure 8C). Afterward, the strain grew with a lower growth rate on gluconate and 2-ketogluconate, which are both produced in the periplasm as intermediates of the glucose metabolism in *P. putida*. This diauxic growth behavior was also observed during investigation of the induction time point for the inducible expression system, but considerably later in the batch phase. At the end of the fermentation, a rhamnolipid titer of about 1.5 g/L was achieved and a slight enrichment of the product in the foam phase was observed over the whole fermentation (Figure 8A). The rhamnolipid titer is 38% higher compared to the shake flask experiment with the flagellum knockout strain. Although an exact comparison is difficult due to the lower rhamnolipid titers in Figure 6, the decisive point is that production in reactors is not worse than in flasks. At the end of the batch, the foam tank was full of foam, because over time a very dry and stable foam was formed in the upper part of the tank, which could not be pumped back efficiently. However, with this bioreactor setup, a product-to-substrate yield of 0.13 g_RL_/g_substrate_ was obtained, which is 30% higher compared to the shake flask experiment. The product-to-biomass yield was 0.59 g_RL_/g_CDW_. Furthermore, a space time yield of 0.16 g_RL_/L/h was reached over the entire fermentation time.

This bioreactor system shows that a scale-up of the rhamnolipid production process is possible without the usage of antifoaming agents or additional equipment during batch operation. By simply pumping back the collapsed foam, it was possible to control the excessive foam formation during the process. This will be a decisive challenge for fed-batch fermentations with higher rhamnolipid concentrations, where additional methods for foam control have to be established.

### Process Development for Subsequent LCA

For the subsequent evaluation of environmental impacts, process chains had to be designed. Consequently, all previously obtained results were integrated and used for the development of a process on a technical scale. For the experiments carried out, the carbon source used was glucose, which is suited best for quantitative lab-scale experiments. However, for industrial production of commodity chemicals, glucose is too expensive. Sustainable substrates considered here instead are sugar beet molasses and sugar beet pulp, as they are by-products of sugar production from sugar beets, which are a major agricultural product in North Rhine-Westphalia (Germany). Thus, two theoretical processes were designed.

**FIGURE 9:**
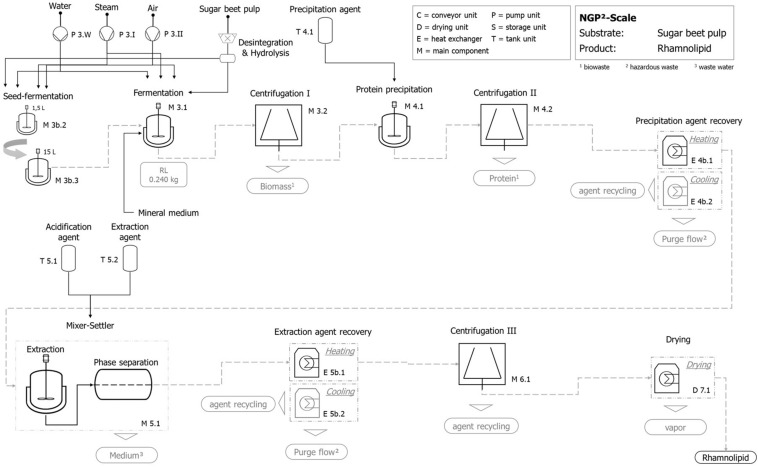
Exemplary qualitative flowchart of the technical scale process for rhamnolipid production with hydrolyzed sugar beet pulp as substrate.

The process shown in Figure 9 represents both studied and implemented process chains RL_I and RL_II, which differ by the used substrate (molasses, sugar beet pulp, both regionally available). Besides the specific throughput of substrate and its allocation coefficient, the technical difference of both process chains is limited to the storage and preparation stage (Supplementary Figure 2). While molasses can be used without preparation, the delivered sugar beet pulp needs to be hydrolyzed (e.g., enzymatically) to increase the accessibility of directly digestible sugar molecules.

The fermentation step includes the production and operation of the fermentation tank as well as the production and operation of the aeration module. The preconnected seed fermentation is included by a two-step tank layout (1.5 and 15 L) and is represented by its production and operation. Furthermore, centrifugation for the separation of fermentation broth and solid shares follows, whereby the utilization of separated biomass (e.g., recycling to process) is not included but factored by a suitable disposal option.

Product isolation and purification include protein precipitation followed by centrifugation, where the precipitation agent (acetone) is added in a volume ratio (agent:broth) Ψ = 1:1 to the fermentation broth. A subsequent precipitation agent recovery by a heating and cooling unit is followed by the extraction stage. The mixer–settler unit is operated after the infeed of the protein-free fermentation broth, using an extraction agent (ethyl acetate, Ψ = 1:5) and an acidification agent amounting to 0.1% (v/v). The emerging heavy aqueous phase without rhamnolipids [except 5% (v/v) losses] is in principle intended for recirculation, which is not included in the present study. Afterward, the contained rhamnolipid is separated by the extraction-agent recovery stage where a combined heating and cooling unit as well as a centrifugation is applied to get an almost finished rhamnolipid product. In a final step, the rhamnolipid is dried again by an appropriate heating unit.

### Environmental Impacts

Based on the developed processes and the related inventory, the implementation in the LCA software and the LCIA for two different process chains was realized. Related to the general key data (Table 3), the different substrate-driven processes show varying starting points. Different sugar contents and preparation steps (hydrolyzation of sugar beet pulp) lead to a different throughput of substrate and further connected input flows (e.g., water and extraction agent). Since the yield and conversion rates do not differ between RL_I and RL_II, these factors do not influence the environmental impact in a process-chain-specific way in the present study.

**FIGURE 10:**
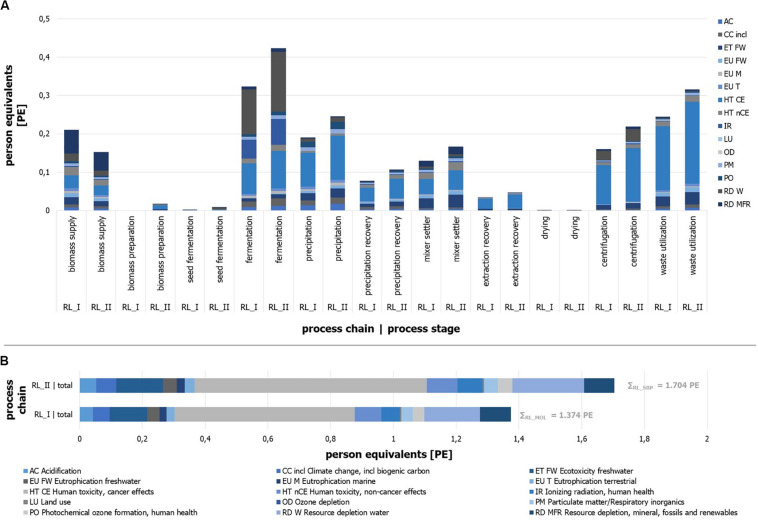
Person equivalents per impact category (ILCD) for process stages of process chain RL_I and RL_II. Absolute number of person equivalents per impact category (ILCD) for process stages of process chain. **(A)** RL_I and **(B)** RL_II.

The implemented process chain RL_II (sugar beet pulp) causes an environmental impact of 1.70 PE, while the use of molasses results in 1.37 PE per kg of product (Figure 10B). Under the assumption of any inaccuracies that may exist, these results can be presumed as slightly different. Distinct differences between the two process chains can be identified by a view on the impact categories.

It can be seen that the impact of process chain RL_I is mainly represented by the impact categories of “Human toxicity, cancer effects” (HT CE), “Ecotoxicity freshwater” (ET FW), and “Resource depletion water” (RD W). Impact generating and mainly (>80%) influencing process modules can be named with the “sugar beet production and processing” (biomass supply), the ethyl acetate production (extraction stage), and acetone purge flow utilization as well as the acetone production (precipitation stage), the fermentation tank production, and the compressed air production (fermentation) for RL_I.

The results of RL_II (Figure 10B) illustrate similar impact contributions by the categories HT CE, RD W, and ET FW. The biomass supply, the treatment of acetone purge flow, and the production of ethyl acetate and acetone, the fermentation tank production, and the compressed air production and electricity supply for the fermentation are the main impacting process modules. The quantitative differences of some individual impact categories are given in several cases by more or less significant deviations.

Besides the identification of specific environmental impact sources, the distribution of contributions in relation to the defined process stages is determined and expressed in PE per process stage (Figure 10A). Reliable derived remarks can be found by the comparison of two stage results for different substrates, but less by the comparison of different process stages due to the randomly defined boundaries of each stage. The results show that the main sources of the molasses-driven process chain impacts can be found in the stages fermentation, waste utilization, and biomass supply for RL_I. Process chain RL_II is dominated by the fermentation, the waste utilization, the precipitation, and the extraction stages.

Furthermore, it can be stated that single impact categories show different shares in the same process stage depending on the process chain (e.g., EU Tof waste utilization or CC of fermentation). In general, it can be seen that most process stages (10 of 11) have larger impacts for RL_II, which can be traced back to the higher throughput due to different dilutions by water. The most impressive example for this is the seed fermentation stage, which is almost five times larger in comparison to RL_I. Most process stages like the drying unit or the precipitation recovery stage illustrate result in the same range for both process chains. On the other hand, RL_I shows one stage with a significantly larger impact. As one (impact-related) decisive process stage, the biomass supply shows the largest impact for RL_I and is 1.4 times higher than for RL_II. Reasons can be found in the parameter of sugar content of substrate, the water-substrate-ratio of the fermentation step, and different economic allocation factors (see goal and scope definition).

## Discussion

In a multidisciplinary approach, we were able to evaluate rhamnolipid production using recombinant *P. putida* KT2440. Regarding the development of producer strains, we were able to optimize the expression cassette to achieve a very stable genome-integrated inducible expression system and engineer the bacterial chassis cell to almost double rhamnolipid titers after the deletion of the flagellar machinery or the PHA-synthesis operon. Regarding lab-scale process engineering we optimized the induction time point and learned that titers can be increased by 18% when inducing early, found an optimal minimal medium by comparing different recipes allowing up to 30% higher rhamnolipid titers, and identified an optimal minimal medium with regard to reduced interphase formation in liquid–liquid extraction. In subsequent upscaling experiments, we were able to meet the performance in shake flasks despite the excessive foaming in aerated fermenters. Based on the achieved results, we further conceptualized a technical-scale process (disregarding the foam challenges for now) and carried out a life-cycle impact assessment identifying the major contributors to the environmental impact.

### Strain Engineering for Robust Production of Heterologous Secondary Metabolites

*Pseudomonas putida* KT2440 has been developed into a platform for recombinant rhamnolipid biosynthesis whereby plasmid-based expression was usually utilized with as strong synthetic constitutive promoters (Loeschcke and Thies, 2020). In a comparative analysis of three production strains harboring expression cassettes with different modes of activation, we observed that inducible production of rhamnolipid biosynthesis outcompeted the constitutive production in terms of strain stability. Remarkably, the plasmid-bearing strain showed the lowest biosurfactant titers despite the by far strongest accumulation of transcript copies. Here, transcript copy numbers after 24 h might even be underestimated in the constitutively expressing strains if mutations accumulated in the population that caused failure in *rhl* gene transcription (e.g., in the promoter region) as suggested by the stability experiment. We consider the exploration of population heterogeneity as an interesting target for further studies. Importantly, maximal transcription appears not necessarily beneficial for product accumulation in this context. This is in line with reports on comparably poor performance of the highly processive T7 polymerase in terms of product levels of an expressed biosynthetic pathway in *P. putida* or *E. coli* (Wittgens, 2013; Domröse et al., 2015; Calero et al., 2016; Du et al., 2017). The salicylate-inducible expression system was previously successfully applied for accumulation of bioactive phenazines in *P. putida* (Schmitz et al., 2015; Bator et al., 2020b) and may, thus, represent a promising extension of the existing toolbox for recombinant natural product biosynthesis. The high selection pressure apparently exerted by rhamnolipids is to some extent astonishing, as *P. putida* is known to resist high concentrations in the medium (Wittgens et al., 2011) and to rebalance the metabolic fluxes upon rhamnolipid production to maintain the balance of essential metabolites (Tiso et al., 2016). Furthermore, this platform strain is considered as very robust concerning the production of bioactive compounds. For example, constitutive production strains for the bioactive compound prodigiosin appeared very stable achieving the same titers after several passages (Domröse et al., 2019). It seems likely that the observed selection pressure during rhamnolipid production is connected to the biosynthesis rather than the compound’s properties itself. It may be due to a massive drain of lipid precursors or other types of metabolic burden, especially during the early growth phases. This hypothesis is supported by the results from the chassis optimization experiments. When relieving the cell from the metabolic burden effectuated by the synthesis of PHA, rhamnolipid titers increase. This indicates that a metabolic limitation was existent despite the remarkable ability to reroute metabolic fluxes (Sudarsan et al., 2014). Further, energy savings might effectuate freeing up of resources which can be utilized for the production of secondary metabolites as indicated by the results obtained with the flagellum mutant. Likewise, Martinez-Garcia et al. (2014) observed an increased energy charge in flagellum-negative *P. putida*. However, the robust metabolism of this bacterium has been shown to outbalance energy drain to a remarkable extent (Ebert et al., 2011). The increase in rhamnolipid titers can thus not be merely explained by an increased energy availability.

A direct consequence of the burden the rhamnolipid synthesis poses on the cell’s metabolism could be observed when looking at the genetic stability of the integrated rhamnolipid synthesis genes. This might also be causing the fluctuating productivity of the recombinant wild-type rhamnolipid producers (700 mg/L vs. 500 mg/L in this study). Using the newly developed inducible expression system should supersede these shortcomings and thus represent an ideal system for biotechnological applications.

While the engineered strains showed beneficial traits regarding rhamnolipid production, they were impaired by the usage of the strong, yet unstable constitutive expression system. For further evaluation and especially for longer cultivations (fed-batch or continuous), the metabolic improvements should be combined with the inducible expression system, which showed clear benefits in terms of genetic stability.

### Process Engineering Approaches for Enhancing Rhamnolipid Production

The presented results regarding liquid–liquid extraction are in basic accordance with our previous results (Biselli et al., 2020). We could show that interphase formation could be reduced by the utilization of a minimal medium within the fermentation process. Despite the fact that interphase formation was reduced compared to our previous results, no increase in the recovery of rhamnolipids in the organic phase could be observed. However, reduced interphase formation is beneficial with regard to the process control within a mixer–settler device.

These findings reveal a new target for strain engineering: Extracellular proteins in the supernatant. Assuming that proteins, which lead to interphase formation within the liquid–liquid extraction and thereby strongly reduce the recovery of the product in the organic solvent, are secreted by the cells and are not the result of lysis, metabolic engineering might provide an appropriate solution. The identification of the main proteins in the interphase might lead to strategies to avoid their formation. Apart from improving the efficiency of downstream processing, this could also lead to a further reduction of metabolic demand and result in enhanced rhamnolipid production.

A major obstacle in the widespread industrial use of microbial biosurfactants is the high production cost compared to conventionally produced crude-oil-based surfactants. One example of microbially produced surfactants that have made to jump to industrial-scale production is the sophorolipids (Henkel et al., 2017). The main production host is *Starmerella bombicola*, which is known to feature high titers of extracellular sophorolipids of above 100 g/L (Daniel et al., 1998). These high values are mainly achieved due to the very low solubility of sophorolipids in water. This entails two crucial advantages: (1) The cost and effort for downstream processing are significantly reduced, and (2) foaming does not occur (Van Bogaert et al., 2007).

However, high titers have also been reported in rhamnolipid production with the wild-type producer *P. aeruginosa*. Titers of about 40 g/L have been reached (Müller et al., 2010), mainly facilitated by the usage of hydrophobic carbon sources such as sunflower oil, which act as defoamers. However, using these substrates complicates the downstream processing, as a stable emulsion is formed if quantities of the substrate remain unused after the fermentation (Marchant and Banat, 2012).

The usage of recombinant *P. putida* KT2440 strains circumvents this downside as the metabolic preferred carbon sources are water-soluble carbohydrates (mainly sugars). Unfortunately, without the second organic phase, foaming becomes a severe challenge. In fact, it has recently been shown that within less than 2 min after inoculation of the bioreactor, the foaming was already intense enough to drive out almost the complete fermentation broth. In a fed-batch fermentation, Beuker et al. (2016a) were able to reach 15 g/L rhamnolipids, however, under the heavy use of antifoaming agents (15 g/L was added).

Fortunately, some solutions to the presented issues have been developed. Already some decades ago, foam fractionation has been identified as a suitable method to cope with the heavy foaming and at the same time enable a pre-purification step, increasing the product concentration in the broth (Siemann-Herzberg and Wagner, 1993). This concept has recently been refined and coupled to direct foam adsorption to collapse the foam and capture the rhamnolipids (Anic et al., 2017). Still, these approaches were burdened by cell loss via foam entrainment. Cells are dragged along by the foam because of hydrophobic proteins on the cell surface “sticking” into the air bubbles. Recently, it has been shown that by eliminating these proteins, cell loss can significantly be reduced (Blesken et al., 2020).

Two recent approaches are again based on the *in situ* use of a second organic phase. In a comprehensive solvent screening, identified ethyl decanoate as suitable extraction solvent for *in situ* product removal (ISPR). The trick is a pH-sensitive partition coefficient, which enables the researchers to completely avoid foaming over the whole fermentation time while reaching a concentration of above 5 g/L rhamnolipids calculated back in the aqueous phase. A second approach used again a combination of strain and process engineering. Bator et al. (2020a) adapted *P. putida* to the usage of ethanol as carbon source and developed a lab-scale process in which ethanol was added to a foam collection bottle to collapse the foam. The formed solution containing the collapsed foam and ethanol was recycled back into the fermenter. This concept facilitated the accumulation of more than 5 g/L of rhamnolipids.

A third approach is based on the usage of specific membranes for aeration. These membranes are designed to only allow diffusion of gasses. Oxygen is thus provided without the formation of bubbles. This is an effective way of avoiding foam formation. In a fed-batch fermentation, titers of above 2 g/L rhamnolipids could thus be reached without the formation of foam (Bongartz et al., in preparation).

These examples show how strain engineering can also contribute to challenges ostensibly only solvable by process engineering. Insights like these underline the need for multidisciplinary research to iterate results between the different research areas and obtain more relevant results.

### Life-Cycle Assessment

The presented LCIA results show quantitatively slightly different environmental impacts (summarized and normalized) in both process chains. An identification of the more advantageous process chain in terms of the used substrate can be cautiously indicated with molasses based on these results. It can be stated that the impact reasons are inside and outside the area of influence. In summary, it can be concluded that the environmental impact clearly depends on certain components of the process chain and the influence of substrate choice is limited in the present setting.

The interpretation of results is limited because of missing comparability to other surfactants, for instance. The chosen scale is suitable to illustrate impact-, energy-, or material-intensive process stages or modules, and the more advantageous variant of process design. Moreover, this scale is usable for further sensitivity analyses to study the systems’ behavior in relation to the systems’ environmental impact. Assuming a type of “environmental economy of scale” and larger yields for complex substrates, a significant reduction of environmental impacts is expected in an industrial-scale model. When combining all favorable parameter changes (optimization of process modules, increasing yield, etc.), it can be assumed that an order of magnitude of the environmental impact comparable to that of conventional surfactants can be achieved.

For the comparison to similar processes and products, with which the own process must potentially compete, the chosen scale should be oriented toward the available data of other process chains. With regard to conventional surfactants, this means that the industrial scale is most likely preferable. The brief history of LCAs related to biosurfactants allows both conclusions, namely, that technical scale as well as industrial scale may be fitting. Summed up, it is getting clear that the definition of intended goals of the LCA study of (bio-)surfactants becomes the decisive factor, whose scale has to be taken into account. A further influencing variable is the provided data, which form the backbone of following design and implementation. A key question to be answered is what changes would appear in a “real” upscale and how these can be considered adequately. The scale-up based on technical-scale data should be realized by a continued iterative process. To get a holistic impression of the system designed sustainability, it should be in mind that also economic and social dimensions of sustainability shall be considered.

## Conclusion

The integrative concept of this study revealed a beneficial combination of inputs from microbiology, bioprocess technology, and downstream processing completed by a life-cycle analysis based on a model process. This enabled the development of optimized stable and safe production strains and downstream strategies for rhamnolipid biosurfactants and the identification of key parameters to be addressed in continued studies with regard to an industrial-scale process based on the results presented here. This study shows in a first step the advantage of comprehensive integration of different research undertakings from multiple disciplines for a common goal and may serve as an incentive for the intensification of multidisciplinary research projects to advance biotechnological processes for microbial biosurfactants and beyond.

## Data Availability Statement

All datasets generated for this study are included in the article/Supplementary Material. The sequence of plasmid pYTSK01K_0G7 was submitted to GenBank with the accession no. MT522186.

## Author Contributions

TT, NI, SKu, AB, AS, IB, and ST designed the experiments. NI, SKu, AS, IB, TK, SKr, and A-LW performed the experimental work. TT, NI, SKu, AS, TK, and A-LW analyzed the data. TT, NI, AB, AS, ST, and TK drafted the manuscript. TT, NI, ST, AL, and K-EJ conceived the research concept. SKu, IB, AL, PZ, AJ, K-EJ, JB, and LB revised the manuscript. All authors read and approved the final manuscript.

## Conflict of Interest

LB and TT declare that they are inventors of three related patents. (1) LB, F. Rosenau, S. Wilhelm, A. Wittgens, TT, “Means and methods for rhamnolipid production” HHU Düsseldorf University, TU Dortmund University, 2013 (WO 2013/041670 A1), (2) LB, B. Küpper, E. M. del Amor Villa, R. Wichmann, C. Nowacki, “Foam adsorption” TU Dortmund University, 2013 (WO 2013/087674 A1), and (3) LB, TT, A. Germer, “Extracellular production of designer hydroxyalkanoyloxy alkanoic acids with recombinant bacteria” RWTH Aachen University, 2015 (WO2017006252A1). The remaining authors declare that the research was conducted in the absence of any commercial or financial relationships that could be construed as a potential conflict of interest.
