# Supplementary material for: Integration of Genetic and Process Engineering for Optimized Rhamnolipid Production Using Pseudomonas putida
Source: Front Bioeng Biotechnol. 2020 Aug 20;8:976. doi: 10.3389/fbioe.2020.00976 (PMC7468518; doi:10.3389/fbioe.2020.00976)
Supplement: Supplementary file 1 [file Data_Sheet_1.PDF]

<u>**Supplementary Material to**</u>

"Integration of genetic and process engineering for optimized rhamnolipid production using *Pseudomonas putida*" by Tiso *et al.*

1.1 Oligonucleotides

Supplementary Table 1. Oligonucleotides used within this work.

Name	Direction	Used for	Sequence
**MaW05**	fwd	*phaG*-KO	gctcggtacccggggatcctCAGGATCGGCCGCGAGAAG
**MaW06**	rev	*phaG*-KO	agtcgatgacGCGGCTCGGCGCCTTGTA
**MaW07**	fwd	*phaG*-KO	gccgagccgcGTCATCGACTCCTGGCGC
**MaW08**	rev	*phaG*-KO	tgcatgcctgcaggtcgactACACCGTACCGTTCTCGAC
**MaW09**	fwd	Sequencing *phaG*-KO	tcaggctgaggatgaaa
**MaW10**	rev	Sequencing *phaG*-KO	cgctacctgatgctgtacaag
**CISL_NagR**	fwd	pYTSK10K_0G7_*rhlAB*	CGGCGAACAGTATAAGCAGATTCGACTCACCAGTCAATATGGGTTTGCTGGAAGCTTATG
**PnagAa_BCD2**	rev	pYTSK10K_0G7_*rhlAB*	TAGAAAACCTCCTTAGCATGATTAAGATGTTTCAGTACGAAAATTGCTTTCATTGTTGATCTCCTTTTTAAGTGAACTTGGGCCGGTACCTCCTGTTTCC
**BCD2_PA-rhlAB**	fwd	pYTSK10K_0G7_*rhlAB*	ACATCTTAATCATGCTAAGGAGGTTTTCTAATGCGGCGCGAAAGTCTG
**PA-rhlAB_I-SceI_CISR**	rev	pYTSK10K_0G7_*rhlAB*	AATAGCGCGAGATTGAGTCGATACCCAGAG*ATTACCCTGTTATCCCTA*TCAGGACGCAGCCTTC
**CISR_eYFP**	fwd	pYTSK40K_1G7_*rhlAB*	CAATTACTCGCGACTTCCTCAATACGTGCTGCAAAACTTTAGAAGGAGATATACCATGGTGAGCAAGGGCGAGGAG
**eYFP_HB-R-rv**	rev	pYTSK40K_1G7_*rhlAB*	TCTGGGTTAAGCGTGTCCTTACACAACAGCACAAGTAAGCTTACTTGTACAGCTCGTCCATG
**Seq_yTREX_Reporter**	fwd	Sequencing pYTSK40K	CTCAATCTCGCGCTATTGTG
**Seq_yTREX_Reporter**	rev	Sequencing pYTSK40K	CCATGGACGCGTAGTCGTAG
**Seq_yTREX_Cluster**	fwd	Sequencing pYTSK10K	AGTGGCTCGTTACCTCGTTC
**Seq_yTREX_Cluster**	rev	Sequencing pYTSK10K	GAGGAAGTCGCGAGTAATTG
**PA-rhlB_fw-RT**	fw	qRT-PCR *rhlB*	CGCTGTTCGACGGCAGTATC
**PA-rhlB_rv-RT**	rev	qRT-PCR *rhlB*	AGGGCCATGGCGTAGAAGTC

1.2 Media

The minimal media used in the experiments are listed here.

M9 medium (Sambrook *et al.* 1989), was modified for RAMOS experiments as follows: 16 g/L glucose, 0.1 M MOPS buffer pH 7, 5 g/L $(NH_4)_2SO_4$, 7.52 g/L $Na_2HPO_4 \times 2\ H_2O$, 3 g/L KH_2PO4, 0.5 g/L NaCl, 0.25 g/L $MgSO_4 \times 7\ H_2O$, 0.15 g/L $CaCl_2 \times 2\ H_2O$, 1 mL/L trace element solution

(4.73 g/L $FeCl_3 \times 6\ H_2O$, 1.5 g/L $MnCl_2 \times 4\ H_2O$, 1.87 g/L $ZnSO_4 \times 7\ H_2O$, 0.3 g/L H_3BO_3, 0.25 g/L $Na_2MoO_4 \times 2\ H_2O$, 0.15 g/L $CuCl_2 \times 2\ H_2O$, 0.84 g/L $Na_2EDTA \times 2\ H_2O$).

Adapted mineral salt medium (Hartmans *et al.* 1989), here called Delft, with the standard phosphate buffer capacity increased 3-fold (11.64 g/L K_2HPO_4, 4.89 g/L, NaH_2PO_4, 2 g/L $(NH_4)_2SO_4$, 10 mg/L EDTA, 100 mg/L $MgCl_2 \times 6\ H_2O$, 2 mg/L $ZnSO_4 \times 7$ H2O, 1 mg/L $CaCl_2 \times 2\ H_2O$, 5 mg/L $FeSO_4 \times 7\ H_2O$, 0.2 mg/L $Na_2MoO_4 \times 2\ H_2O$, 0.2 mg/L $CuSO_4 \times 5\ H_2O$, 0.4 mg/L $CoCl_2 \times 6\ H_2O$, 1 mg/L $MnCl_2 \times 2\ H_2O$).

M9 medium (Sambrook *et al.* 1989) (12.8 g/L $Na_2HPO_4 \times 2\ H_2O$, 3 g/L KH_2PO_4, 0.5 g/L NaCl, 1.0 g/L NH_4Cl, 2 mM $MgSO_4$) was supplemented for media comparison experiments with 2 mL/L US trace-elements solution (82.81 mL/L 37 % fuming HCl, 4.87 g/L $FeSO_4 \times 7\ H_2O$, 4.12 g/L $CaCl_2 \times 2\ H_2O$, 1.50 g/L $MnCl_2 \times 4\ H_2O$, 1.87 g/L $ZnSO_4 \times 7\ H_2O$, 0.30 g/L H_3BO_3, 0.25 g/L $Na_2MoO_4 \times 2\ H_2O$, 0.15 g/L $CuCl_2 \times 2\ H_2O$, 0.84 g/L $Na_2EDTA \times 2\ H_2O$).

Wilms MOPS (5.0 g/L $(NH_4)_2SO_4$, 0.5 g/L NH_4Cl, 3 g/L K_2HPO_4, 2 g/L Na_2SO_4, 41.9 g/L 4-Morpholinpropansulfonsäure (MOPS), 0.5 g/L $MgSO_4 \times 7H_2O$, 0.01 g/L Thiaminhydrochlorid, 3 mL/L trace-element solution (0.54 g/L $ZnSO_4 \times 7\ H_2O$, 0.31 g/L $CuSO_4 \times 5\ H_2O$, 0.3 g/L $MnSO_4 \times H_2O$, 0.54 g/L $CoCl_2 \times 6\ H_2O$, 41.76 g/L $FeCl_3 \times 6\ H_2O$, 1.98 g/L $CaCl_2 \times 2\ H_2O$, 33.39 g/L EDTA (Titriplex III)) (Wilms *et al.* 2001, Scheidle *et al.* 2007).

ModR (22 g/L KH_2PO_4, 2.6 g/L (NH_4)2HPO_4, 1.4 g/L $MgSO_4 \times 7\ H_2O$, 0.87 g/L citric acid, 0.01 g/L $FeSO_4 \times 7\ H_2O$, 5 g/L glucose, 10 mL/L trace element solution 2, pH 6.8) for fermentation (Beuker *et al.* 2016).

1.3 RT-qPCR

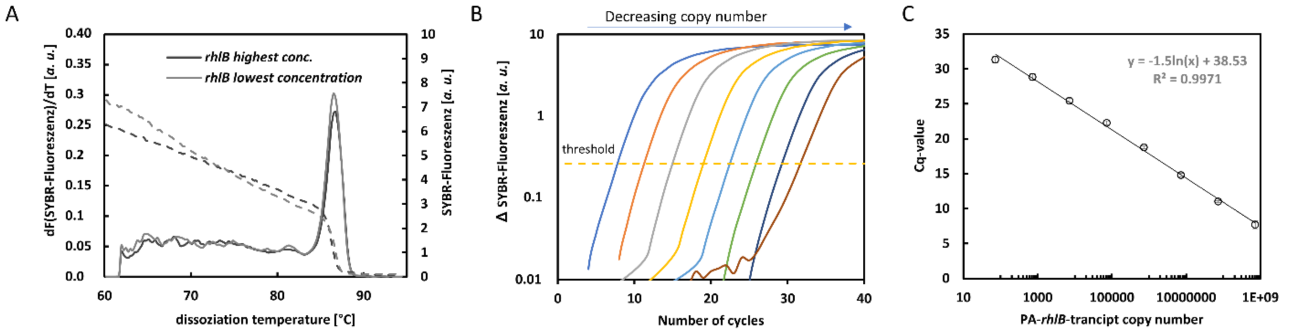

Supplementary Figure 1: Quality control and transcript copy number calibration of *rhlB* qRT-PCR. A Validation of the PCR specificity using the primer PA-*rhlB*_fw-RT, PA-*rhlB*_rv-RT and the template pYTSK40K_1G7_*rhlAB*. Depicted are exemplarily the dissociation curves and their first derivatives for the reactions with the highest applied template concentration (ca. 70,000,000 copies) as well as for the lowest (ca. 700 copies). B Courses of normalized SYBR Green fluorescence (ΔRn) during the qPCR with the different template dilutions. C Calibration plot of the Cq value (number of cycles at threshold fluorescence) versus template copy number. The equation derived from the

regression (y = -1.5ln(x)+38.53, with R^2= 0.997) was used for the determination of *rhlB* transcript copy numbers in the biological samples.

1.4 Flow chart of the technical scale process for rhamnolipid production with molasses as substrate

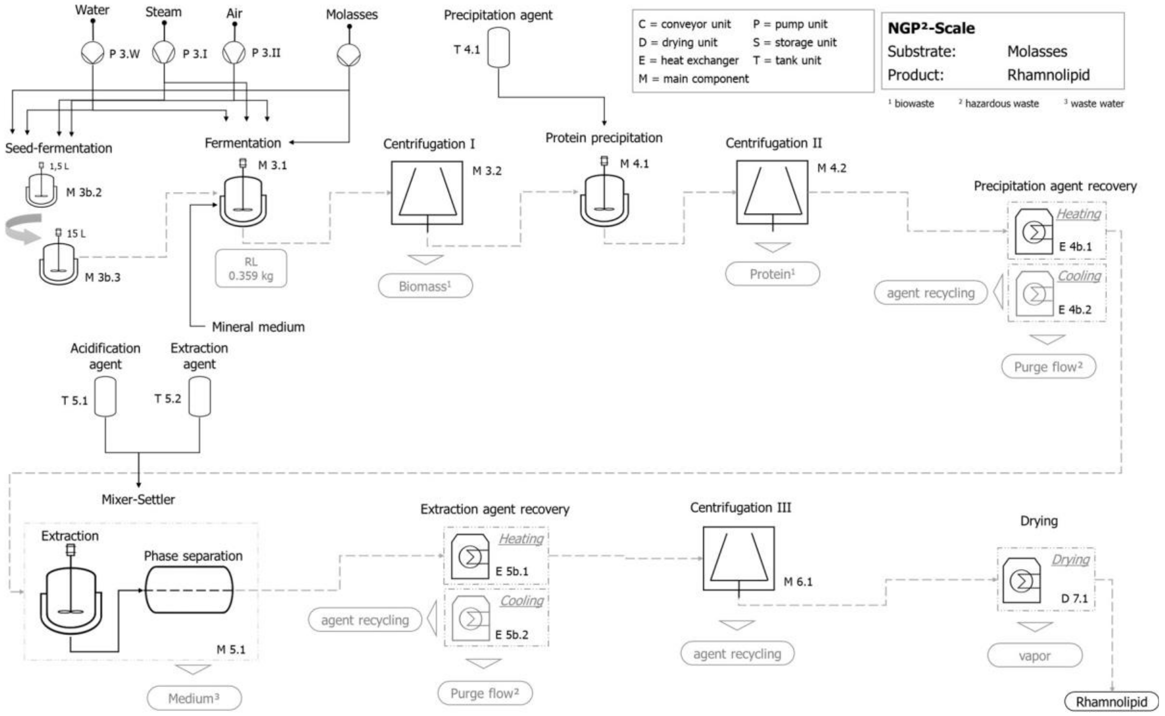

Supplementary Figure 2: Exemplary qualitative flow chart of the technical scale process for rhamnolipid production with molasses as substrate.

1.5 Early induction leads to higher productivity

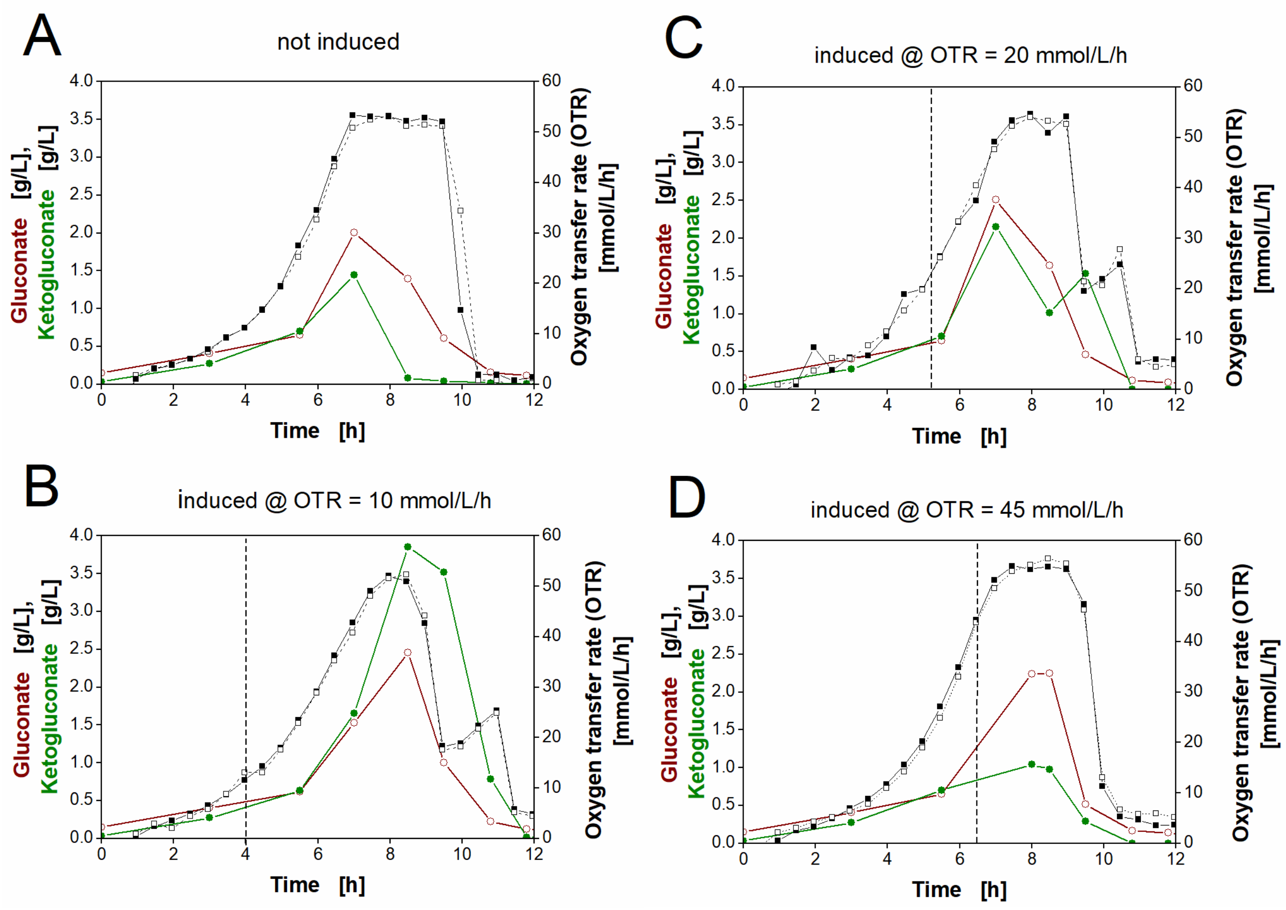

Supplementary Figure 2: Physiological studies of *P. putida* KT2440 SK40 for variation of time of the timepoint of induction with salicylate. Detailed physiological data for gluconate (open circles), ketogluconate (closed circles) and oxygen transfer rate (squares) at different times of induction. The dashed vertical line marks the induction time point. Induction with 2 mM salicylate after 4 h (B), 5.3 h (C) and 6.5 h (D) and non-induced culture (A). Cultivation conditions: 250 mL shake flasks with 10 mL filling volume, M9 minimal medium (17 g/L glucose, 0.1 M MOPS buffer, pH 7) at a shaking frequency of 250 rpm with a shaking diameter of 50 mm and an initial optical density of 0.1.

1.6 Goal and scope definition for LCA

The environmental impact is expressed by 16 impact categories and their specific category units as "Acidification" in Mole of H^+ equivalents, "Climate change excl biogenic carbon" in kg CO_2 equivalents, "Climate change midpoint, incl biogenic carbon" in kg CO_2 equivalents, "Ecotoxicity freshwater" in CTUe, "Eutrophication freshwater" in kg P equivalents, "Eutrophication marine" in kg N equivalents, "Eutrophication terrestrial" in Mole of N equivalents, "Human toxicity, cancer effects" in CTUh, "Human toxicity, non-cancer" effects in CTUh, "Ionizing radiation, human health" in kBq U235 equivalents, "Land use" in kg C deficit equivalents, "Ozone depletion" in kg CFC-11

equivalents, "Particulate matter/Respiratory inorganics" in kg PM2.5 equivalents, "Photochemical ozone formation midpoint, human health" in kg NMVOC equivalents, "Resource depletion water" in m^3 equivalents, and "Resource depletion, mineral, fossils and renewables" in kg Sb equivalents. These categories are taken from the above mentioned ILCD recommendations.

Supplementary Table 2: List of 16 impact categories and related specific units according to the ILCD recommendations

Impact category	Specific unit
Acidification midpoint	[Mole of H+ eq.]
Climate change midpoint, excl biogenic carbon	[kg CO_2 eq.]
Climate change midpoint, incl biogenic carbon	[kg CO_2 eq.]
Ecotoxicity freshwater midpoint	[CTUe]
Eutrophication freshwater midpoint	[kg P eq.]
Eutrophication marine midpoint	[kg N eq.]
Eutrophication terrestrial midpoint	[Mole of N eq.]
Human toxicity midpoint, cancer effects	[CTUh]
Human toxicity midpoint, non-cancer effects	[CTUh]
Ionizing radiation midpoint, human health	[kBq ^{235}U eq.]
Land use midpoint	[kg C deficit eq.]
Ozone depletion midpoint	[kg CFC-11 eq.]
Particulate matter/Respiratory inorganics midpoint	[kg $PM_{2.5}$ eq.]
Photochemical ozone formation midpoint, human health	[kg NMVOC eq.]
Resource depletion water, midpoint	[m^3 eq.]
Resource depletion, mineral, fossils and renewables, midpoint	[kg Sb eq.]

References

Beuker J, Steier A, Wittgens A, Rosenau F, Henkel M and Hausmann R (2016). "Integrated foam fractionation for heterologous rhamnolipid production with recombinant *Pseudomonas putida* in a bioreactor." AMB Express **6**(1): 11.

Hartmans S, Smits JP, Vanderwerf MJ, Volkering F and Debont JAM (1989). "Metabolism of styrene oxide and 2-phenylethanol in the styrene-degrading *Xanthobacter* strain 124X." Applied and Environmental Microbiology **55**(11): 2850-2855.

Sambrook J, Maniatis T and Fritsch EF (1989). Molecular cloning: A laboratory manual. Cold Spring Harbor, NY, Cold Spring Harbor Laboratory Press.

Scheidle M, Klinger J and Büchs J (2007). "Combination of on-line pH and oxygen transfer rate measurement in shake flasks by fiber optical technique and Respiration Activity MOnitoring System (RAMOS)." Sensors (Basel, Switzerland) **7**(12): 3472-3480.

Wilms B, Hauck A, Reuss M, Syldatk C, Mattes R, Siemann M and Altenbuchner J (2001). "High-cell-density fermentation for production of L-*N*-carbamoylase using an expression system based on the *Escherichia coli rhaBAD* promoter." Biotechnology and Bioengineering **73**(2): 95-103.
